# Higher fibrosis-4 index is associated with 2-year cardiovascular events in Vietnamese adults with type 2 diabetes mellitus: a prospective cohort study

**DOI:** 10.3389/fgstr.2026.1973311

**Published:** 2026-10-07

**Authors:** Quoc Bao Vu, Hong Minh Cong Vo, Xuan Linh Tran, Tuan Vu Nguyen, Thi Khanh Tuong Tran

**Affiliations:** 1 Internal Medicine Department, Pham Ngoc Thach University of Medicine, Ho Chi Minh, Vietnam; 2 Nhan Dan Gia Dinh Hospital, Ho Chi Minh, Vietnam

**Keywords:** cardiovascular events, FIB-4, liver fibrosis, MACE, MAFLD, transient elastography, type 2 diabetes, Vietnam

## Abstract

**Background:**

Cardiovascular disease is a major extrahepatic outcome in type 2 diabetes mellitus (T2DM) and metabolic fatty liver disease, but prospective data integrating transient elastography and the fibrosis-4 index (FIB-4) with observed cardiovascular events remain limited in Southeast Asia.

**Methods:**

We conducted a prospective cohort study of adults with T2DM consecutively recruited at a Vietnamese tertiary hospital from June 2023 through January 2025 and followed for up to 24 months. Hepatic steatosis and liver stiffness were assessed by FibroScan^©^, and metabolic dysfunction-associated fatty liver disease (MAFLD) was defined using the Asian Pacific Association for the Study of the Liver framework. Expanded major adverse cardiovascular events (MACE) were the principal time-to-event outcome. Sparse-event associations were estimated with Firth-penalized Cox regression.

**Results:**

Of 452 patients screened, 301 consented and 284 entered the baseline cohort; 280 contributed follow-up. At baseline, MAFLD was present in 72.5% and significant liver fibrosis (liver stiffness measurement ≥7.0 kPa) in 23.9%. During 544.92 person-years, 11 expanded MACE and 5 conventional MACE occurred, corresponding to incidence rates of 20.19 (95% CI 10.08–36.12) and 9.18 (95% CI 2.98–21.41) per 1,000 person-years. In the main Firth model adjusted for age and MAFLD, each increase of 1 interquartile range (IQR; 0.696 units) in FIB-4 was associated with expanded MACE (adjusted hazard ratio 1.56, 95% CI 1.09–1.99; p = 0.022), whereas MAFLD was not statistically significant (adjusted hazard ratio 3.51, 95% CI 0.79–33.09; p = 0.105); the point estimate was above unity but imprecise because of the limited number of events.

**Conclusion:**

Higher FIB-4 was associated with short-term cardiovascular events in adults with T2DM. Given the small number of events, these findings require confirmation in larger multicenter cohorts.

## 1 Introduction

Type 2 diabetes mellitus (T2DM) is increasing worldwide and carries substantial cardiovascular and liver-related morbidity. Global diabetes prevalence among adults aged 20–79 years is projected to reach 12.2% by 2045 (1). Steatotic liver disease is highly prevalent in T2DM. A recent global meta-analysis of more than 2.2 million adults with T2DM estimated a pooled non-alcoholic fatty liver disease (NAFLD)/metabolic dysfunction-associated steatotic liver disease (MASLD) prevalence of 65.3%; in studies from 2016–2021, the estimate was 68.8% (2). Among biopsy-selected patients with T2DM and NAFLD/MASLD, approximately two-thirds had non-alcoholic steatohepatitis (NASH)/metabolic dysfunction-associated steatohepatitis (MASH), although this estimate may be higher than in the general T2DM population because biopsy cohorts are enriched for patients at greater liver risk (2). Studies using transient elastography suggest that approximately one quarter of adults with T2DM have significant liver fibrosis (2). Fibrosis stage is a key determinant of liver-related prognosis. In a long-term cohort of biopsy-proven NAFLD, Hagström et al. found progressively greater risk of severe liver disease with increasing fibrosis stage, whereas NASH did not add prognostic information beyond fibrosis stage (3). Contemporary liver guidelines emphasize non-invasive fibrosis assessment because fibrosis risk is more closely associated with clinically important outcomes than steatosis alone (4–7).

The nomenclature of metabolic fatty liver disease has evolved. The international multisociety framework now uses MASLD, whereas the Asian Pacific Association for the Study of the Liver (APASL) retains metabolic dysfunction-associated fatty liver disease (MAFLD) as a positive diagnostic framework that permits coexistence with other liver disease etiologies (4, 5). This study was designed and classified using the APASL MAFLD framework; we therefore retain the term MAFLD rather than retrospectively relabeling participants as MASLD.

Cardiovascular disease is a leading cause of morbidity and mortality in T2DM and a major extrahepatic outcome of metabolic fatty liver disease. Meta-analyses and large longitudinal cohorts have shown higher cardiovascular event rates among people with NAFLD, MAFLD, or MASLD, although estimates vary according to disease definition, metabolic burden, fibrosis severity, and cardiovascular endpoint composition (8–13). In people with T2DM, the Rio de Janeiro Cohort Study further showed that greater liver stiffness measured by transient elastography was associated with subsequent cardiovascular outcomes (14).

The fibrosis-4 index (FIB-4) is a simple blood-based index derived from age, aspartate aminotransferase, alanine aminotransferase, and platelet count. Although developed for liver fibrosis risk stratification, longitudinal studies suggest that FIB-4 also carries prognostic information for cardiovascular events and mortality in people with obesity and/or T2DM (15, 16). More recently, Song et al. reported that higher FIB-4 was associated with all-cause and cardiac mortality in two large prospective cohorts of patients with coronary heart disease and diabetes or prediabetes (17). These observations are clinically relevant but require cautious interpretation because FIB-4 is not a direct measure of fibrosis, and age is included in its formula.

Tuong et al. used FibroScan^©^ in 307 Vietnamese adults with T2DM and documented a high burden of fatty liver disease and fibrosis (18). In an earlier cross-sectional analysis from the initial recruitment phase of the same research program, involving 291 adults with T2DM, significant liver fibrosis was associated with carotid atherosclerosis and increased carotid intima-media thickness after multivariable adjustment, with similar or stronger associations in the MAFLD subgroup (19). That analysis addressed subclinical vascular disease at baseline and could not determine whether liver-related markers were associated with subsequent clinical cardiovascular events. Separately, Dang et al. reported that FIB-4 correlated with systematic coronary risk evaluation 2 (SCORE2)/systematic coronary risk evaluation 2–older persons (SCORE2-OP) estimated 10-year cardiovascular risk in Vietnamese adults with MAFLD, but their study was cross-sectional and did not assess incident cardiovascular events (20).

Prospective Vietnamese cardiovascular outcome data have only recently begun to emerge in other high-risk populations. In 168 patients with stable chronic obstructive pulmonary disease, Doan et al. prospectively observed 24 MACE during 12 months and found baseline MAFLD associated with a higher hazard of incident MACE (21). Although informative, that cohort differs in underlying disease and baseline cardiovascular risk.

To our knowledge, no Vietnamese cohort has combined baseline transient elastography with prospectively recorded cardiovascular events in adults with T2DM. We recruited such a cohort and followed it for up to 24 months, examining the associations of FIB-4, MAFLD, and liver stiffness measurement (LSM)-defined significant liver fibrosis with incident cardiovascular events. Outcomes included conventional major adverse cardiovascular events (MACE), comprising cardiovascular death, non-fatal myocardial infarction, and non-fatal ischemic or hemorrhagic stroke, and expanded MACE, which additionally included hospitalization for heart failure, transient ischemic attack, hospitalization for unstable angina, peripheral arterial events, and coronary or peripheral revascularization. Expanded MACE was the principal endpoint; conventional MACE was analyzed separately.

## 2 Materials and methods

### 2.1 Study design and setting

This prospective cohort study was conducted at Nhan Dan Gia Dinh Hospital, Ho Chi Minh City, Vietnam, a tertiary hospital in Ho Chi Minh City, Vietnam. Participants were consecutively recruited from 1 June 2023 through 31 January 2025, underwent standardized baseline clinical, laboratory, and liver assessments, including transient elastography at enrollment, and were followed for cardiovascular outcomes for up to 24 months. Administrative study follow-up ended on July 31, 2026. During the initial recruitment phase, baseline data from an overlapping subset of participants were used in a previously published cross-sectional analysis of significant liver fibrosis and carotid atherosclerosis (19). Recruitment for the present prospective cohort continued after that cross-sectional analytic dataset had been assembled, and the longitudinal analysis used cardiovascular eligibility and baseline data-completeness requirements specific to the present research question. The two analytic samples therefore overlap but are not identical. The previous publication reported baseline liver and carotid findings only; no prospective MACE outcomes from the present analysis were reported previously. Reporting follows the STROBE framework for cohort studies (22).

### 2.2 Participants and recruitment

Adults aged 18 years or older with T2DM attending hospital-based outpatient clinics in General Internal Medicine, Endocrinology, Gastroenterology, and Cardiology were screened consecutively throughout the predefined recruitment period. Patients were not enrolled if they declined participation; had a history of conventional MACE before baseline; had New York Heart Association class III or IV heart failure; had an estimated glomerular filtration rate ≤30 mL/min/1.73 m2; had acute hepatitis with alanine aminotransferase or aspartate aminotransferase ≥150 U/L; had ascites, hepatocellular carcinoma, secondary liver malignancy, or metastatic cancer; or were pregnant or breastfeeding. Ascites was excluded for technical reasons related to transient elastography and was not intended as a criterion for excluding decompensated cirrhosis as a disease category. Inclusion in the longitudinal analytic cohort additionally required a technically reliable FibroScan^©^ examination and sufficient prespecified baseline information for the cardiovascular analyses.

### 2.3 Baseline assessment

At enrollment, study staff recorded age, sex, known diabetes duration, smoking, alcohol use, physical activity, comorbidities, medication use, weight, height, waist circumference, and blood pressure. Laboratory testing included complete blood count, aspartate aminotransferase (AST), alanine aminotransferase (ALT), creatinine, fasting glucose, glycated hemoglobin (HbA1c), triglycerides, total cholesterol, low-density lipoprotein (LDL) cholesterol, high-density lipoprotein (HDL) cholesterol, hepatitis B surface antigen when indicated, and anti-HCV testing when indicated.

### 2.4 Liver phenotyping and FIB-4

Transient elastography was performed with a FibroScan^©^ 530 Compact system (Echosens, Paris, France) using the M probe. Participants fasted for at least 3 h. Measurements were obtained from the right hepatic lobe through an intercostal space. An examination was considered reliable when at least 10 valid measurements were obtained, the success rate was ≥60%, and the interquartile range/median ratio was <30% for both controlled attenuation parameter (CAP) and liver stiffness measurement (LSM).

The steatosis and stiffness thresholds were prespecified in the study protocol and were unchanged from the baseline liver-vascular analyses reported from the same research program (19). Hepatic steatosis was defined as CAP ≥234 dB/m, corresponding to S1 or greater in the categorical scheme used in prior Vietnamese FibroScan^©^ work in adults with T2DM (S0 ≤233, S1 234–269, S2 270–300, and S3 ≥301 dB/m) (18). This CAP threshold was retained for continuity with the local protocol and should not be interpreted as a universal histologic cutoff. LSM-defined significant liver fibrosis was prespecified as LSM ≥7.0 kPa. The 7.0-kPa threshold was supported by biopsy-validated transient elastography data in NAFLD, in which it provided the best overall cutoff for ≥F2 fibrosis, and it was also used in the prior Vietnamese T2DM FibroScan^©^ study (18, 23). Because all participants had T2DM, APASL criteria classified every participant with hepatic steatosis as having MAFLD (4). The MAFLD framework did not require exclusion of coexisting viral hepatitis or alcohol exposure. Additional measurement details are provided in **Supplementary Methods S1**.

FIB-4 was calculated as (age [years] × AST [U/L])/(platelet count [10^9^/L] × √ALT [U/L]) and was analyzed primarily as a continuous variable. FIB-4 and LSM-defined significant liver fibrosis were not entered simultaneously into the same multivariable model. For Kaplan–Meier analyses, FIB-4 was additionally categorized using age-adjusted thresholds. In participants aged <65 years, low, intermediate, and high categories were defined as <1.3, 1.3–2.67, and >2.67, respectively. Following the age-adjustment principle recommended in contemporary AASLD and EASL guidance, the lower cutoff was increased to 2.0 for participants aged ≥65 years; in this age group, categories were <2.0, 2.0–2.67, and >2.67 (5, 6). These categories were developed for liver-fibrosis risk stratification rather than cardiovascular prediction and were used here only for visualization of event patterns.

### 2.5 Cardiovascular outcomes, verification, and follow-up

Conventional MACE comprised cardiovascular death, non-fatal myocardial infarction, and non-fatal stroke (ischemic or hemorrhagic). Expanded MACE comprised conventional MACE plus hospitalization for heart failure, transient ischemic attack, hospitalization for unstable angina, and peripheral arterial events or coronary/peripheral revascularization. Definitions were prespecified and aligned, where applicable, with contemporary cardiovascular outcome standards (24); detailed definitions are provided in **Supplementary Table S1**.

Participants were contacted approximately every 3 months, and outpatient visits and hospital admissions were reviewed for potential cardiovascular events. Each suspected event was verified against source medical documentation and confirmed by a physician in the relevant specialty. All but one qualifying event occurred at the study hospital. One participant was hospitalized at another hospital for unstable angina and underwent percutaneous coronary intervention with coronary stent implantation; this event was verified from the hospital discharge summary and written documentation of stent placement. No cardiovascular endpoint was based solely on participant self-report.

Analyses used time to first qualifying event. If more than one qualifying cardiovascular component occurred during the same clinical episode, the participant contributed one event to the time-to-first-event analysis. Time at risk extended from enrollment to the first qualifying event, last known follow-up, completion of 24 months, or the administrative study end on 31 July 2026, whichever came first. Participants who remained event-free but had not completed 24 months when the study closed were administratively censored at their last observed follow-up.

### 2.6 Statistical analysis

Categorical variables are summarized as counts and percentages. Continuous variables are presented as mean and standard deviation when approximately normally distributed and as median with interquartile range (IQR) otherwise. For Cox regression reporting, FIB-4 hazard ratios were rescaled to one interquartile-range increase in the overall time-to-event cohort (IQR = 0.696 units; Q1 = 0.939 and Q3 = 1.635). The same 0.696-unit scaling factor was used in subgroup analyses to facilitate direct comparison of effect estimates; this linear rescaling does not alter model fit or p-values. Crude incidence rates were calculated per 1,000 person-years with 95% confidence intervals. Expanded-MACE-free survival by MAFLD status was described using Kaplan–Meier methods and compared with the log-rank test. Exploratory Kaplan–Meier analyses were also performed across the three age-adjusted FIB-4 categories. Because these categories are ordered, the principal categorical comparison in the full cohort used the log-rank test for trend; pairwise log-rank comparisons were reported descriptively in the **Supplementary Material**. The same age-adjusted FIB-4 categories were examined within the MAFLD subgroup using standard log-rank comparisons as an exploratory subgroup visualization.

Candidate covariates were prespecified on clinical grounds and included age, sex, diabetes duration ≥10 years, hypertension, MAFLD, HbA1c <7%, LDL cholesterol ≥2.6 mmol/L, and one liver-related fibrosis variable. Depending on the model, the liver-related fibrosis variable was either continuous FIB-4 or LSM-defined significant liver fibrosis (LSM ≥7.0 kPa). LSM was evaluated both as a continuous variable and as LSM-defined significant liver fibrosis (≥7.0 kPa) in univariable Firth Cox models for expanded and conventional MACE. Bayesian model averaging (BMA) was used only for expanded MACE as an exploratory assessment of model uncertainty rather than as an automated rule for selecting a single final model. Two separate BMA spaces were analyzed: one containing continuous FIB-4 and one containing LSM-defined significant liver fibrosis, with the other seven candidate covariates identical. Each space therefore contained eight optional predictors (256 possible predictor subsets). Both BMA datasets contained all 280 participants and all 11 expanded MACE. Posterior inclusion probabilities (PIPs) were reported as measures of the stability of evidence across competing models. The model search retained 99 models in the FIB-4 space and 81 in the LSM space. Full BMA settings are provided in **Supplementary Methods S2** and **Supplementary Table S2**.

Because only 11 expanded MACE and 5 conventional MACE occurred, univariable and multivariable Cox models used Firth-penalized likelihood to reduce small-sample and separation bias. To avoid unstable high-dimensional adjustment, the core expanded-MACE model was restricted to age and FIB-4, and the main inferential model added MAFLD status to address the principal liver phenotype of interest. Because age is incorporated in the standard FIB-4 formula, a sensitivity analysis omitted age only as a separate covariate while retaining FIB-4 calculated with its standard age-containing formula; no age-free modification of FIB-4 was constructed. Additional sensitivity analyses repeated the core analysis with conventional Cox regression and used LSM-defined significant liver fibrosis in place of FIB-4 where appropriate. Collinearity was assessed with variance inflation factors (VIF), and the incremental information contributed by age and by FIB-4 was evaluated with penalized likelihood-ratio tests. Proportional-hazards assumptions were assessed using scaled Schoenfeld residuals from the corresponding conventional Cox model. When evidence of non-proportionality was observed, exploratory time-varying analyses were performed using a FIB-4 × log(time/18 months) interaction and a complementary piecewise analysis split at 12 months. Given the small number of events, these analyses were considered exploratory and were not used to replace the prespecified main model. To quantify the influence of individual cardiovascular events on the FIB-4 estimates, leave-one-event-out post-hoc sensitivity analyses were performed by sequentially omitting each participant with an event and refitting the corresponding Firth Cox model. For the post-hoc leave-one-event-out analysis, the age-, FIB-4-, and MAFLD-adjusted main model was refitted for expanded MACE, whereas the age- and FIB-4-adjusted model was refitted for conventional MACE; the fixed overall-cohort FIB-4 IQR of 0.696 units was retained in all refitted models. The MAFLD subgroup was analyzed separately, and conventional MACE analyses were considered exploratory. Two-sided p < 0.05 was considered statistically significant, while PIPs were not interpreted as frequentist significance tests. Analyses were performed with IBM SPSS Statistics 27.0.1.0 and R 4.6.0. Additional details of the categorical and piecewise analyses are provided in **Supplementary Methods S3**.

### 2.7 Ethics

The study was conducted in accordance with the Declaration of Helsinki and Good Clinical Practice principles. The protocol was approved by the ethics committees of Nhan Dan Gia Dinh Hospital, Ho Chi Minh City, Vietnam (approval No. 75/NDGD-HDDD). Written informed consent was obtained from participants according to the approved protocol.

## 3 Results

### 3.1 Cohort assembly and baseline characteristics

From 1 June 2023 through 31 January 2025, 452 adults with T2DM were screened; 301 provided written informed consent and 151 declined participation. Seventeen consenting participants were excluded before baseline analysis: 3 due to acute hepatitis with AST and/or ALT ≥150 U/L, 13 due to substantial missing baseline laboratory data, and 1 due to an unreliable FibroScan^©^ examination. The baseline cohort therefore comprised 284 participants. Four had no post-baseline follow-up information, leaving 280 participants for time-to-first-event analyses (**Figure 1**). In all, 13 event-free participants had 18–23 months of follow-up when the study closed and were administratively censored at their last observed contact. Median follow-up was 24 months (interquartile range, 24–24 months), and total follow-up was 544.92 person-years.

**Figure 1:**
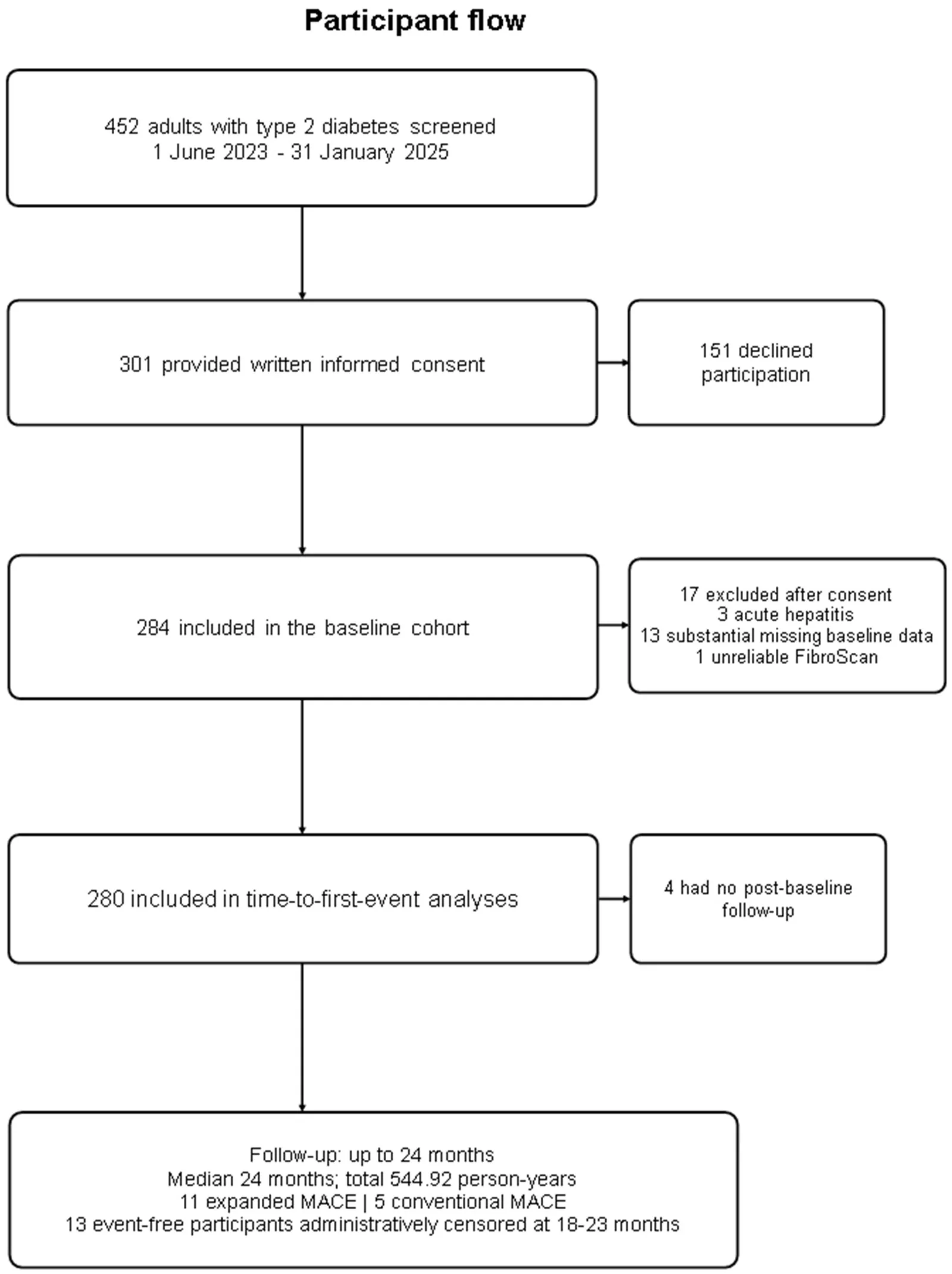
Participant flow from screening through the cardiovascular time-to-first-event cohort. MACE, major adverse cardiovascular events.

At baseline, mean age was 59.1 ± 10.7 years and 42.6% were men. Median known diabetes duration was 6.0 years (IQR, 1.0–11.0 years). MAFLD was present in 206 of 284 participants (72.5%). Median FIB-4 was 1.2 (IQR, 0.9–1.6), median LSM was 5.5 kPa (IQR, 4.4–6.9 kPa), and 68 participants (23.9%) met the study definition of significant liver fibrosis. Baseline characteristics are summarized in **Table 1**. Full baseline characteristics by MAFLD status are provided in **Supplementary Table S3**.

**Table 1:** Baseline characteristics by MAFLD status (n = 284).

Characteristic	Overall	No MAFLD	MAFLD
Age, years	59.1 ± 10.7	60.8 ± 10.5	58.5 ± 10.7
Male sex, n (%)	121 (42.6)	39 (50.0)	82 (39.8)
Body mass index (BMI), kg/m2	24.7 ± 3.7	23.1 ± 2.8	25.3 ± 3.8
Overweight/obesity, n (%)	195 (68.7)	40 (51.3)	155 (75.2)
Abdominal obesity, n (%)	234 (82.4)	52 (66.7)	182 (88.3)
Known diabetes duration, years	6.0 (1.0–11.0)	6.5 (1.0–13.0)	5.0 (1.0–10.3)
Diabetes duration ≥10 years, n (%)	97 (34.2)	31 (39.7)	66 (32.0)
Hypertension, n (%)	205 (72.2)	55 (70.5)	150 (72.8)
Dyslipidemia, n (%)	262 (92.3)	66 (84.6)	196 (95.1)
Smoking, n (%)	42 (14.8)	16 (20.5)	26 (12.6)
Significant alcohol use, n (%)	34 (12.0)	10 (12.8)	24 (11.7)
Chronic hepatitis B, n (%)	27 (9.5)	6 (7.7)	21 (10.2)
Chronic hepatitis C, n (%)	7 (2.5)	3 (3.8)	4 (1.9)
HbA1c, %	7.0 (6.4–8.3)	6.9 (6.3–8.4)	7.1 (6.5–8.3)
LDL cholesterol ≥2.6 mmol/L, n (%)	151 (53.2)	34 (43.6)	117 (56.8)
FIB-4	1.2 (0.9–1.6)	1.3 (1.0–1.7)	1.2 (0.9–1.6)
CAP, dB/m	265.1 ± 58.0	195.1 ± 37.1	291.7 ± 39.3
Liver stiffness, kPa	5.5 (4.4–6.9)	4.8 (4.1–6.0)	5.7 (4.5–7.3)
Significant liver fibrosis (LSM ≥7.0 kPa), n (%)	68 (23.9)	12 (15.4)	56 (27.2)
Statin use, n (%)	177 (62.3)	50 (64.1)	127 (61.7)
SGLT2 inhibitor use, n (%)	15 (5.3)	4 (5.1)	11 (5.3)

Values are mean ± SD, median (interquartile range), or n (%). No hypothesis-test p-values are shown because **Table 1** is descriptive. CAP, controlled attenuation parameter; LSM, liver stiffness measurement; MAFLD, metabolic dysfunction-associated fatty liver disease; SGLT2, sodium-glucose cotransporter-2.

### 3.2 Cardiovascular events and incidence rates

Eleven of 280 participants experienced expanded MACE (3.9%); 5 participants experienced conventional MACE (1.8%). Observed expanded MACE spanned cardiovascular death, stroke, hospitalization for heart failure, transient ischemic attack, unstable angina, and coronary or peripheral arterial revascularization. Components occurring during the same clinical episode were counted as one event in the time-to-first-event analysis. The incidence rate was 20.19 per 1,000 person-years (95% CI 10.08–36.12) for expanded MACE and 9.18 per 1,000 person-years (95% CI 2.98–21.41) for conventional MACE (**Table 2**).

**Table 2:** Cardiovascular outcomes and incidence rates during follow-up (n = 280).

Outcome	Participants with event, n	Person-years	Incidence per 1,000 person-years (95% CI)
Expanded MACE	11	544.92	20.19 (10.08–36.12)
Conventional MACE	5	544.92	9.18 (2.98–21.41)

Time-to-first-event analysis. Multiple qualifying components during the same clinical episode were counted once. Expanded MACE included conventional MACE plus hospitalization for heart failure, transient ischemic attack, hospitalization for unstable angina, and qualifying coronary/peripheral arterial events or revascularization.

### 3.3 Primary analyses for expanded MACE

In univariable Firth Cox analyses, age (hazard ratio [HR] 1.11 per year, 95% CI 1.04–1.19; p < 0.001), diabetes duration ≥10 years (HR 3.15, 95% CI 1.003–11.127; p = 0.049), and FIB-4 (HR 1.72, 95% CI 1.28–2.12; p = 0.002) were associated with expanded MACE. MAFLD had an imprecise estimate and was not statistically significant (HR 2.74, 95% CI 0.65–25.42; p = 0.192). Neither LSM-defined significant liver fibrosis (HR 1.86, 95% CI 0.53–5.83; p = 0.315) nor continuous LSM (HR 1.03 per 1-kPa increase, 95% CI 0.92–1.07; p = 0.371) was associated with expanded MACE. Full univariable estimates are shown in **Supplementary Table S4**.

In the core Firth model, age (adjusted HR [aHR] 1.10, 95% CI 1.03–1.17; p = 0.005) and FIB-4 (aHR 1.62, 95% CI 1.13–2.07; p = 0.014) were associated with expanded MACE. In the main model adding MAFLD, associations were maintained for age (aHR 1.11, 95% CI 1.03–1.19; p = 0.003) and FIB-4 (aHR 1.56, 95% CI 1.09–1.99; p = 0.022), whereas MAFLD was not statistically significant (aHR 3.51, 95% CI 0.79–33.09; p = 0.105) (**Table 3**). The VIF for age and FIB-4 was 1.20. Penalized likelihood-ratio testing supported incremental information from FIB-4 beyond age (chi-square = 6.02; p = 0.014) and from age beyond FIB-4 (chi-square = 7.95; p = 0.005).

**Table 3:** Firth-penalized cox models for expanded MACE.

Variable	Core model (age + FIB-4)	Main model (age + FIB-4 + MAFLD)	Age-omitted sensitivity (FIB-4 + MAFLD)
Age, per year	1.10 (1.03–1.17); p = 0.005	1.11 (1.03–1.19); p = 0.003	–
FIB-4, per IQR (0.696-unit) increase	1.62 (1.13–2.07); p = 0.014	1.56 (1.09–1.99); p = 0.022	1.68 (1.27–2.07); p = 0.002
MAFLD	–	3.51 (0.79–33.09); p = 0.105	2.72 (0.64–25.23); p = 0.198

Entries are adjusted hazard ratios (95% CI); p-value. FIB-4 estimates are reported per IQR (0.696-unit) increase derived from the overall time-to-event cohort. Core model: age + FIB-4. Main model: age + FIB-4 + MAFLD. The age-omitted model omits age only as a separate covariate; FIB-4 retains its standard formula, which includes age. All estimates are sparse-event estimates and should be interpreted cautiously.

When age was omitted as a separate covariate, with FIB-4 retained in its standard age-containing form, the association remained similar (aHR 1.68, 95% CI 1.27–2.07; p = 0.002). Conventional Cox regression yielded estimates in the same direction (age aHR 1.12, 95% CI 1.04–1.20; FIB-4 aHR 1.49, 95% CI 1.09–2.06).

In leave-one-event-out sensitivity analyses, the adjusted HR for FIB-4 remained above 1.0 in all 11 refitted expanded-MACE models and ranged from 1.22 to 1.63, compared with 1.56 in the full cohort. Ten of the 11 analyses retained p < 0.05. In one refitted model, omission of an individual event attenuated the FIB-4 estimate to 1.22 (95% CI 0.54–2.40; p = 0.600), indicating sensitivity of the magnitude and precision of the estimate to individual-event deletion (**Supplementary Table S5**).

### 3.4 Bayesian model averaging and model uncertainty

Both BMA analyses used n = 280 with 11 expanded MACE. In the FIB-4 model space, age had the highest PIP (96.3%), followed by FIB-4 (71.5%), MAFLD (65.3%), HbA1c <7% (58.3%), LDL cholesterol ≥2.6 mmol/L (39.3%), male sex (23.9%), diabetes duration ≥10 years (23.1%), and hypertension (22.5%). In the alternative LSM model space, age had a PIP of 100.0%, MAFLD 71.3%, HbA1c <7% 52.3%, LDL cholesterol ≥2.6 mmol/L 37.7%, male sex 28.1%, hypertension 26.7%, diabetes duration ≥10 years 21.1%, and LSM-defined significant liver fibrosis 19.9%. The BMA procedure retained 99 and 81 models, respectively. These results were used to characterize model uncertainty and did not determine the final inferential model; full settings and PIPs are reported in **Supplementary Methods S2** and **Supplementary Table S2**.

### 3.5 Proportional-hazards diagnostics and time-varying analyses

The global Schoenfeld test for the main expanded-MACE model was not statistically significant (p = 0.096), but FIB-4 showed evidence of non-proportional hazards (p = 0.012), whereas age (p = 0.578) and MAFLD (p = 0.953) did not. In an exploratory model using a continuous time interaction, the FIB-4 × log(time/18 months) term was significant (p = 0.025). A complementary piecewise analysis split follow-up at 12 months. Three expanded MACE occurred during the first 12 months and eight thereafter in the full cohort. The piecewise model suggested a stronger FIB-4 association after 12 months, but the formal coefficient representing a discrete change after 12 months was not statistically significant (p = 0.317). Within the MAFLD subgroup, three events occurred during the first 12 months and seven thereafter; the corresponding change term was also not statistically significant (p = 0.169). Profile-likelihood interval estimation was unstable for some piecewise terms. Detailed proportional-hazards and related sensitivity results are provided in **Supplementary Table S6** and **Supplementary Figures S1–S4**. These temporal analyses were therefore considered exploratory and do not establish a 12-month change point; detailed piecewise estimates are provided in **Supplementary Table S7**.

### 3.6 MAFLD-related analyses

Kaplan–Meier analysis suggested lower expanded-MACE-free survival among participants with MAFLD, but the between-group difference was not statistically significant (log-rank test chi-square = 1.978, df = 1; p = 0.160; **Figure 2**).

**Figure 2:**
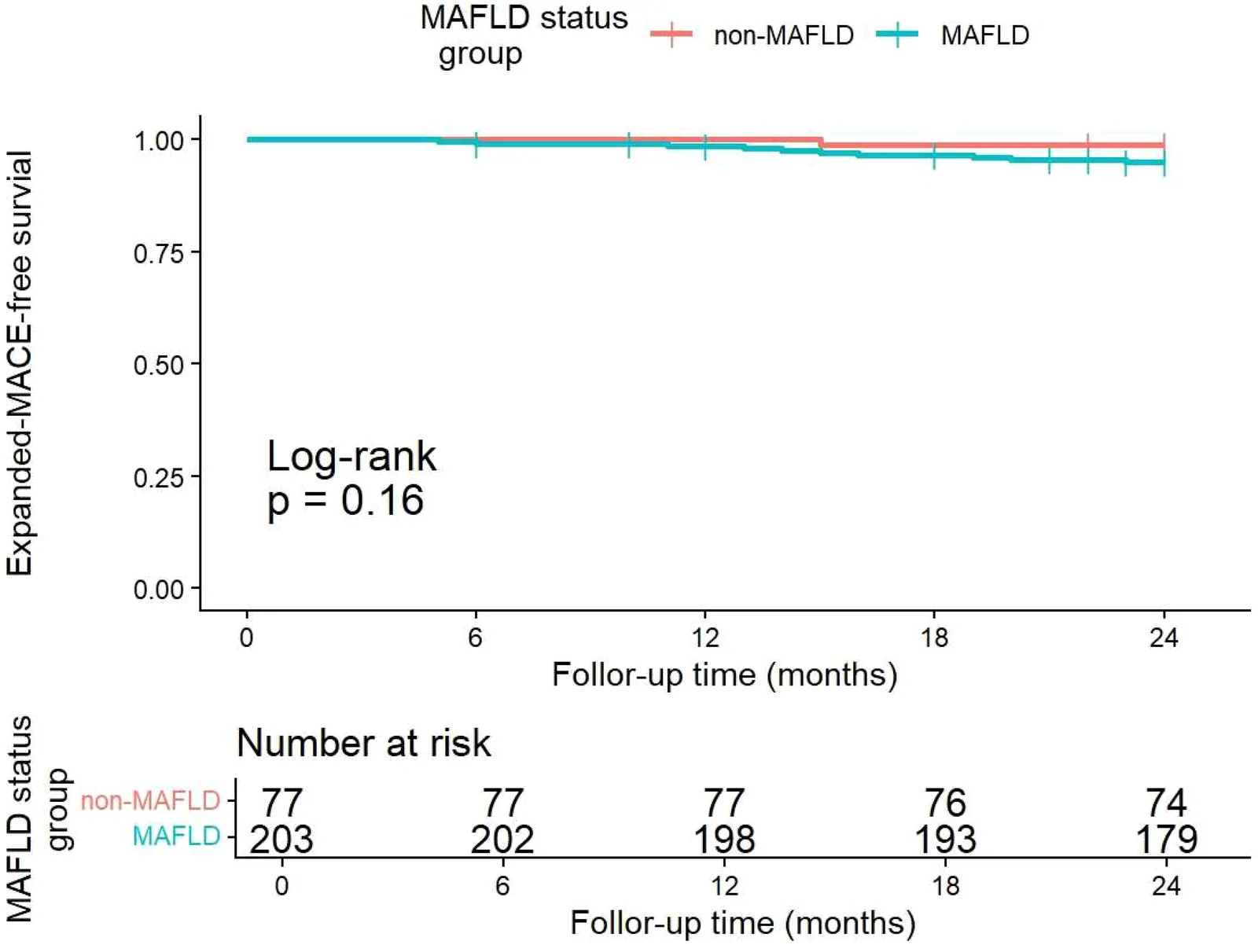
Kaplan–Meier estimates of expanded-MACE-free survival across MAFLD categories. The log-rank test was non-significant (chi-square = 1.978, df = 1; p = 0.160). Expanded MACE occurred in 10/203 (4.93%) and 1/77 (1.30%) participants in MAFLD and non-MAFLD groups, respectively. Censoring marks and numbers at risk are shown.

Within the MAFLD subgroup, 10 expanded MACE occurred over 392.0 person-years (25.51 per 1,000 person-years; 95% CI 12.23–46.91). After mutual adjustment, age (aHR 1.13, 95% CI 1.04–1.22; p = 0.002) and FIB-4 (aHR 1.63, 95% CI 1.17–2.06; p = 0.009) remained associated with expanded MACE. In a model replacing FIB-4 with LSM-defined significant liver fibrosis, significant liver fibrosis was not associated with expanded MACE (aHR 1.01, 95% CI 0.27–3.47; p = 0.994). Detailed expanded-MACE analyses within the MAFLD subgroup are provided in **Supplementary Table S8**.

### 3.7 Exploratory age-adjusted FIB-4 categorical analyses

Among the 280 participants in the time-to-event cohort, the age-adjusted FIB-4 categories contained 191 participants in the low-risk group, 79 in the intermediate-risk group, and 10 in the high-risk group. Expanded MACE occurred in 5/191 (2.6%), 4/79 (5.1%), and 2/10 (20.0%), respectively. Kaplan–Meier analysis showed evidence of an ordered trend toward lower expanded-MACE-free survival across increasing FIB-4 categories (log-rank test for trend chi-square = 5.11, df = 1; p = 0.024; **Figure 3**). For conventional MACE, the corresponding event proportions were 2/191 (1.0%), 2/79 (2.5%), and 1/10 (10.0%); the ordered trend was directionally similar but did not reach statistical significance (chi-square = 3.25, df = 1; p = 0.071; **Supplementary Figure S5**). Because the high-FIB-4 category contained only 10 participants, these categorical analyses were considered exploratory; pairwise comparisons are reported in **Supplementary Table S9**.

**Figure 3:**
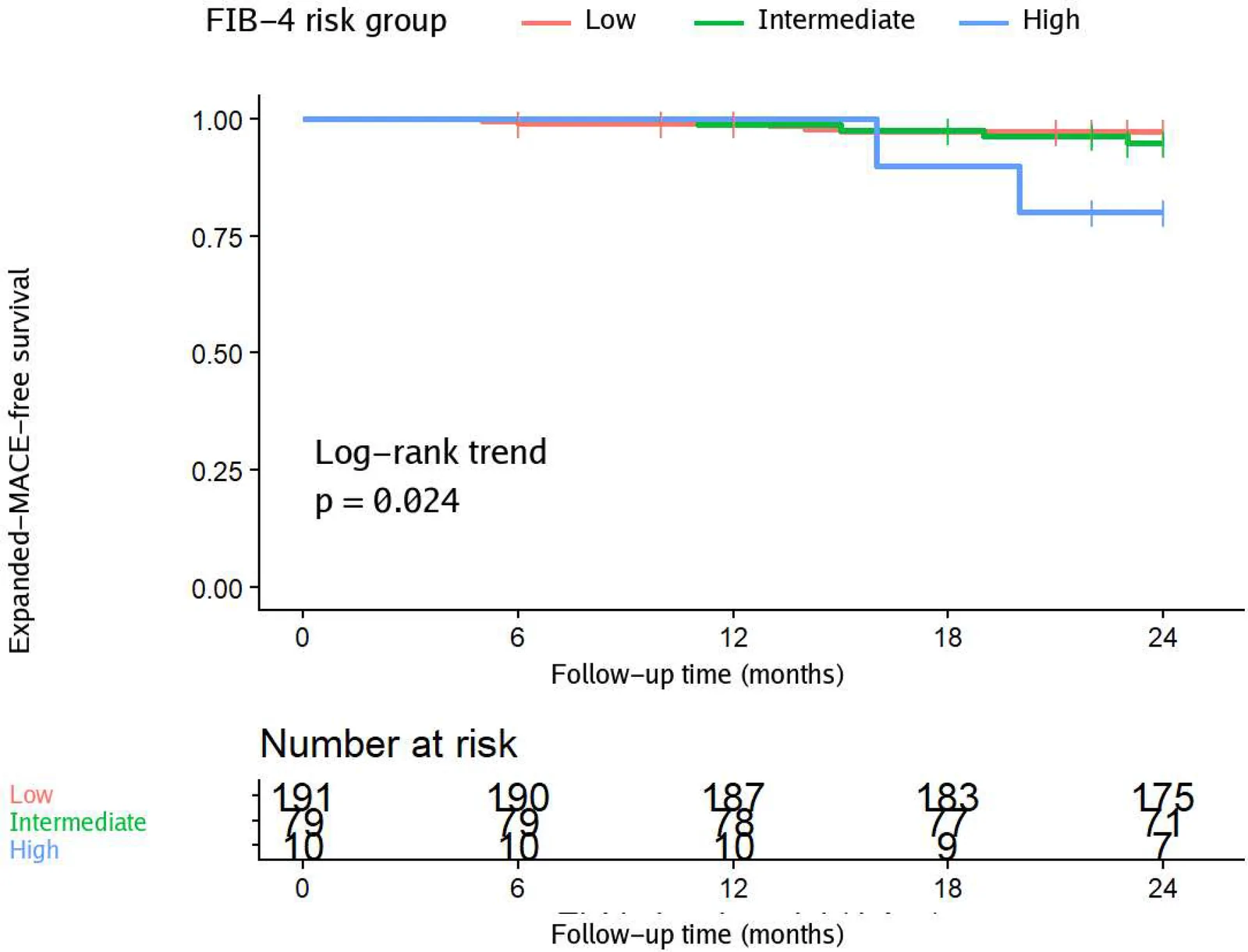
Kaplan–Meier estimates of expanded-MACE-free survival across age-adjusted FIB-4 risk categories. For participants aged <65 years, low, intermediate, and high categories were defined as FIB-4 <1.3, 1.3–2.67, and >2.67, respectively; for participants aged ≥65 years, the lower cutoff was increased to 2.0, yielding categories <2.0, 2.0–2.67, and >2.67. The ordered log-rank test for trend was significant (chi-square = 5.111, df = 1; p = 0.024). Expanded MACE occurred in 5/191 (2.6%), 4/79 (5.1%), and 2/10 (20.0%) participants in the low-, intermediate-, and high-risk groups, respectively. Censoring marks and numbers at risk are shown.

In categorical analyses within the MAFLD subgroup, the age-adjusted FIB-4 strata comprised 139 low-risk, 58 intermediate-risk, and 6 high-risk participants. Kaplan–Meier curves differed across categories for expanded MACE (log-rank p = 0.0026; **Supplementary Figure S6**). A similar separation was observed for conventional MACE (log-rank p = 0.016; **Supplementary Figure S7**). Because only six participants were in the high-risk category and only four conventional MACE occurred within the MAFLD subgroup, these categorical subgroup findings were considered exploratory.

### 3.8 Exploratory conventional MACE analyses

Only 5 conventional MACE occurred. In a two-variable Firth model containing age and FIB-4, FIB-4 remained associated with conventional MACE (aHR 1.81, 95% CI 1.24–2.39; p = 0.007), whereas age did not (aHR 1.08, 95% CI 0.98–1.19; p = 0.129). The conventional Cox estimate for FIB-4 was directionally similar (aHR 1.76, 95% CI 1.23–2.50; p = 0.002). In the MAFLD subgroup, which contained four conventional MACE, the age-adjusted Firth estimate for FIB-4 was 1.87 (95% CI 1.32–2.56; p = 0.003), and conventional Cox regression yielded a similar estimate (aHR 1.87, 95% CI 1.31–2.65; p = 0.001). Continuous LSM was not significantly associated with conventional MACE in univariable Firth Cox analysis (HR 1.05 per 1-kPa increase, 95% CI 0.96–1.08; p = 0.155). These conventional-MACE analyses are exploratory because of the very small event count and are detailed in **Supplementary Tables S10, S11**. In leave-one-event-out analyses for conventional MACE, the age-adjusted FIB-4 estimate remained above 1.0 in all five refitted models and ranged from 1.23 to 1.98, compared with 1.81 in the full cohort. Four of five analyses retained p < 0.05; in one analysis, omission of an individual event attenuated the estimate to 1.23 (95% CI 0.33–3.33; p = 0.720) (**Supplementary Table S5**).

## 4 Discussion

### 4.1 Principal findings

In this prospective cohort of Vietnamese adults with T2DM, higher FIB-4 was associated with an increased risk of cardiovascular events during follow-up. Eleven expanded MACE occurred over 544.92 person-years, corresponding to an incidence rate of 20.19 per 1,000 person-years. After adjustment for age and MAFLD, each 1-IQR increase in FIB-4 was associated with a 56% higher hazard of expanded MACE. Similar associations were observed when age was excluded from the model and when conventional Cox regression was used. Higher FIB-4 was also associated with expanded MACE within the MAFLD subgroup. Although only 5 conventional MACE occurred, the association with FIB-4 was similar for this more restrictive endpoint.

MAFLD was not significantly associated with expanded MACE after adjustment for age and FIB-4, although the point estimate was above unity and 10 of the 11 events occurred among participants with MAFLD. LSM-defined significant liver fibrosis was also not associated with expanded MACE. In contrast, FIB-4 remained significantly associated with expanded MACE, although the small number of events limits comparisons across these liver-related markers.

### 4.2 FIB-4 and cardiovascular risk

The direction of the FIB-4 finding is consistent with larger international longitudinal studies. Anstee et al. reported that higher FIB-4 was associated with subsequent cardiovascular events among more than 44,000 individuals with obesity and/or T2DM (15), while Guan et al. identified associations between FIB-4 and cardiovascular outcomes in adults with diabetes in NHANES (16). More recently, Song et al. showed that higher FIB-4 was associated with all-cause and cardiac mortality after multivariable adjustment in two prospective cohorts of patients with coronary heart disease and diabetes or prediabetes (17). In Vietnam, Dang et al. showed that FIB-4 increased across SCORE2/SCORE2-OP cardiovascular-risk categories and correlated with estimated 10-year cardiovascular risk in adults with MAFLD (20). Their study used calculated risk rather than prospectively observed events. The present study adds prospective evidence from Vietnamese adults with T2DM who had no history of conventional MACE. All participants also underwent baseline transient elastography, allowing FIB-4 and liver stiffness to be evaluated within the same cohort.

FIB-4 should not be interpreted as a direct measure of hepatic fibrosis in this context. Because it incorporates age, AST, ALT, and platelet count, its association with cardiovascular events may reflect information beyond liver fibrosis alone. AST is also not liver-specific and may be influenced by skeletal muscle, so differences in muscle mass or frailty could affect FIB-4 while also being associated with cardiovascular risk.

Age is particularly important because it is both a major cardiovascular determinant and an explicit component of FIB-4. We therefore examined whether the association could be explained largely by the age term. The VIF for age and FIB-4 was 1.20, penalized likelihood-ratio comparisons supported incremental information from each variable beyond the other, and FIB-4 remained significantly associated with expanded MACE when age was deliberately omitted. These analyses make it less likely that the observed association was produced solely by entering age both directly and within the FIB-4 formula. However, they do not establish FIB-4 as an age-independent cardiovascular biomarker, because age remains biologically embedded in the index.

Exploratory Kaplan–Meier analyses using age-adjusted FIB-4 categories showed increasing event frequency across categories. However, only 10 participants were classified as high risk, and these thresholds were developed for liver-fibrosis risk stratification rather than cardiovascular prognosis. Accordingly, the categorical analyses were considered exploratory, whereas continuous FIB-4 was used to characterize the association without assigning cardiovascular significance to liver-derived cutoffs. These data do not establish a cardiovascular FIB-4 threshold or determine whether cardiovascular risk varies meaningfully within the conventional low-risk range.

A similar pattern was observed within the MAFLD subgroup, but only six participants were in the high-risk category, and only four conventional MACE occurred. These findings do not establish a cardiovascular FIB-4 threshold.

### 4.3 MAFLD and cardiovascular events

MAFLD was not significantly associated with expanded MACE after adjustment for age and FIB-4 in this cohort, although the point estimate was above unity and imprecise. In the age- and FIB-4-adjusted Firth model, the estimated hazard was approximately 3.5-fold higher among participants with MAFLD, but the confidence interval was extremely wide; 10 of the 11 expanded MACE occurred in this group. Kaplan–Meier analysis similarly suggested lower event-free survival among participants with MAFLD, although the between-group difference was not statistically significant. The study had limited ability to estimate this association, particularly because MAFLD was present in nearly three-quarters of the cohort and only one expanded event occurred in the non-MAFLD group. Previous longitudinal studies have reported higher cardiovascular risk in NAFLD, MAFLD, or MASLD (8–13), but the present cohort was too small to confirm an independent association. Accordingly, the present data cannot establish or exclude an independent cardiovascular effect of MAFLD.

More recently, Doan et al. followed 168 Vietnamese patients with stable chronic obstructive pulmonary disease for 12 months and reported 24 MACE, with baseline MAFLD associated with a higher hazard of incident events (21). That cohort provides additional prospective evidence linking MAFLD with cardiovascular events in Vietnamese clinical populations but differs substantially from the present cohort in underlying disease, event frequency, endpoint composition, and metabolic risk structure.

For conventional MACE, four of five events occurred among participants with MAFLD, but the univariable hazard estimate was close to unity and highly imprecise. Therefore, any suggestion of greater cardiovascular burden associated with MAFLD in this cohort applies mainly to expanded MACE and should not be generalized to a demonstrated effect on conventional MACE.

### 4.4 Liver stiffness and cardiovascular events

Vietnamese FibroScan data have previously documented a substantial burden of steatotic liver disease and liver fibrosis in adults with T2DM (18). The 23.9% prevalence of LSM-defined significant liver fibrosis in our cohort was close to the 24.6% pooled estimate reported in transient elastography studies of adults with T2DM (2). Despite this substantial fibrosis burden, LSM-defined significant liver fibrosis was not associated with cardiovascular events. This finding was not limited to the prespecified 7.0-kPa threshold. When LSM was analyzed as a continuous variable, it was also not significantly associated with expanded or conventional MACE. In the Rio de Janeiro Cohort Study, Cardoso et al. reported that greater liver stiffness, particularly at LSM >9.6 kPa, was associated with cardiovascular outcomes in patients with T2DM and NAFLD (14). Our prespecified threshold of 7.0 kPa identifies a broader and less advanced fibrosis stage, beginning at approximately F2, and therefore differs from a higher stiffness threshold. However, because continuous LSM was also not associated with cardiovascular events, the difference from the Rio de Janeiro cohort cannot be explained solely by dichotomization at 7.0 kPa. The small number of cardiovascular events and the relatively short follow-up are particularly important. With only 11 expanded and 5 conventional MACE, the study had limited statistical information to estimate the association between liver stiffness and clinical cardiovascular outcomes with precision. The 7.0-kPa cutoff was not selected from cardiovascular outcomes in the present cohort; it was based on prior biopsy-validated NAFLD transient elastography data and had also been applied in Vietnamese adults with T2DM (18, 23). Taken together, the contrasting findings for FIB-4 and LSM suggest that FIB-4 may capture cardiovascular or systemic risk beyond liver fibrosis alone. However, the absence of a significant LSM association may also reflect the small number of events and limited statistical precision.

An earlier cross-sectional analysis from the initial recruitment phase of the same research program included 291 adults with T2DM and found that significant liver fibrosis was associated with carotid atherosclerosis (odds ratio [OR] 2.1) and increased carotid intima-media thickness (OR 2.3) after multivariable adjustment, with similar or stronger associations in the MAFLD subgroup (19). Because that report used an overlapping participant pool, it should not be considered independent replication of the current findings. The two analyses assessed different outcomes and had substantially different statistical information. Subclinical carotid atherosclerosis was substantially more frequent than clinical cardiovascular events and therefore provided much greater statistical information. Moreover, clinical cardiovascular events reflect multiple processes beyond atherosclerotic burden. Given the small number of events, the present findings do not exclude an association between greater liver stiffness and longer-term cardiovascular risk.

### 4.5 Analytical considerations and model uncertainty

Because only 11 expanded MACE occurred, the final inferential models were deliberately parsimonious, and Firth penalization was used to reduce small-sample bias. BMA was used separately to describe uncertainty in covariate selection rather than to choose the final model. The retention of 99 models in the FIB-4 space and 81 in the LSM space indicates substantial uncertainty about model structure.

Within the separate BMA spaces, FIB-4 had a PIP of 71.5% and LSM-defined significant fibrosis had a PIP of 19.9%. These values are not a head-to-head comparison and do not establish superiority of FIB-4. The FIB-4 association was also similar in conventional Cox regression, after exclusion of age, and within the MAFLD subgroup, although all analyses were derived from the same cohort.

For expanded MACE, proportional-hazards diagnostics suggested possible non-proportionality for FIB-4: the global Schoenfeld test was not significant, whereas the variable-specific test for FIB-4 was. A continuous FIB-4 × log(time) interaction was significant, suggesting that the magnitude of the association may vary during follow-up. The complementary 12-month piecewise analysis also suggested a stronger association after the first year, but the formal coefficient for a discrete change after 12 months was not statistically significant in either the full cohort or the MAFLD subgroup, and profile-likelihood interval estimation was unstable for some terms. Thus, the temporal analyses are compatible with a non-constant FIB-4 association but do not establish a biologically meaningful 12-month breakpoint. For conventional MACE, the corresponding time-varying interaction was not statistically significant. With only 11 and 5 events, respectively, these temporal patterns were treated as sensitivity analyses. The principal Cox hazard ratios are best interpreted as average associations over the observed follow-up period.

### 4.6 Clinical implications

FIB-4 is calculated from routinely available clinical variables at essentially no additional cost and is already used in liver-fibrosis risk pathways for people with T2DM and metabolic liver disease (4–7). Our findings, together with recent prospective data linking higher FIB-4 to cardiac mortality in patients with coronary heart disease and diabetes or prediabetes (17), support further evaluation of FIB-4 as an adjunct to established cardiovascular risk assessment. They do not support its use as a stand-alone cardiovascular prediction tool, and whether FIB-4 improves risk discrimination or reclassification remains uncertain.

The 1-IQR estimate represents a fixed 0.696-unit increase in FIB-4, approximately the difference between the 25th and 75th percentiles of the cohort. This continuous association should not be interpreted using conventional low/intermediate/high FIB-4 thresholds, which were developed for liver-fibrosis risk stratification rather than cardiovascular prediction. Although the findings raise the possibility that cardiovascular information may extend across the FIB-4 distribution, the present study does not establish a cardiovascular threshold or a risk gradient within the conventional low-risk range.

Clinically, a high FIB-4 may serve as a flag to review established cardiovascular risk factors more closely, but it should not be interpreted as a validated cardiovascular risk category.

### 4.7 Strengths and limitations

This study has several strengths. The cohort was recruited prospectively within a predefined enrollment window. Baseline assessment was standardized and completed before any outcome occurred; participants were contacted approximately every 3 months, and only four of the 284 participants entering the baseline cohort had no post-baseline follow-up; the time-to-event cohort retained 280 participants and 544.92 person-years.

Hepatic steatosis and liver stiffness were assessed directly by transient elastography rather than inferred from diagnostic codes or metabolic surrogate measures. Cardiovascular endpoints were prespecified and verified against source medical records by a physician in the relevant specialty rather than accepted from participant self-report. All but one qualifying event occurred at the study hospital; the single event treated elsewhere was confirmed from the discharge summary and written documentation of coronary stent implantation. Components arising within the same clinical episode were counted once in the time-to-first-event analysis.

The limitations are substantial. Most importantly, this was a single-center study with only 11 expanded and 5 conventional cardiovascular events. The limited event information constrained the number of covariates that could be estimated reliably and precluded comprehensive conventional multivariable modeling. The leave-one-event-out analysis quantified the influence of individual events on the FIB-4 estimates. For expanded MACE, the FIB-4 estimate remained above unity in all 11 refitted models and remained statistically significant in 10; for conventional MACE, it remained above unity in all five models and statistically significant in four. However, omission of an individual event substantially attenuated the estimate and widened the confidence interval in each endpoint. The direction of association was preserved, but the effect size and statistical significance were sensitive to individual events. Firth penalization reduces small-sample bias, but neither penalization nor sensitivity analysis can compensate for the limited information provided by only 11 expanded and 5 conventional MACE events. Confidence intervals for less frequent exposures, particularly MAFLD, were therefore wide, and the conventional-MACE analyses—especially those restricted to the MAFLD subgroup—are exploratory. In addition, because all participants had T2DM, the presence of hepatic steatosis was sufficient to satisfy the APASL MAFLD definition. This resulted in a high MAFLD prevalence and a relatively small non-MAFLD comparison group. Only one event occurred among participants without MAFLD, substantially limiting the precision with which an independent MAFLD effect could be estimated. Accordingly, the present study cannot establish or exclude an independent cardiovascular effect of MAFLD. The age-adjusted categorical FIB-4 analyses and 12-month piecewise analyses were also exploratory; the high FIB-4 category contained only 10 participants, and some piecewise profile-likelihood intervals did not converge fully.

Selection bias is also possible because 151 of 452 screened patients declined participation. Participants may therefore differ from otherwise eligible patients who did not enroll, limiting generalizability beyond patients willing to undergo detailed assessment at a Vietnamese tertiary hospital. A subset of participants also contributed baseline data to the earlier cross-sectional publication from the same research program. This overlap was disclosed explicitly; the earlier and present analytic samples were not identical, and no prospective MACE outcomes were reported in the previous article; nevertheless, the earlier cross-sectional findings should not be interpreted as independent validation of the present cohort.

Residual confounding remains likely because the small event count precluded simultaneous adjustment for the full spectrum of cardiovascular determinants, kidney-related variables, longitudinal glycemic exposure, and contemporary cardioprotective therapies. Treatment variables measured at baseline are also vulnerable to confounding by indication. The expanded-MACE endpoint increased the amount of event information available during relatively short follow-up but combined outcomes of differing severity and pathophysiology. Conventional MACE was therefore analyzed separately. FIB-4 showed a similar association with this narrower outcome, although only five conventional events occurred.

Finally, BMA estimates depend on the prespecified candidate set and prior assumptions and should therefore be interpreted as exploratory.

## 5 Conclusion

Among Vietnamese adults with T2DM followed for up to 24 months, higher FIB-4 was associated with expanded MACE and showed a similar association with conventional MACE. MAFLD was not significantly associated with expanded MACE, although its estimate was imprecise, and LSM-defined significant liver fibrosis was not associated with short-term cardiovascular events. Given the small number of events and the observed sensitivity of effect estimates to individual-event deletion, these findings should be considered hypothesis-generating. Larger multicenter cohorts are required to determine whether FIB-4 provides prognostic information beyond established cardiovascular risk factors.

## Data Availability

The datasets presented in this article are not readily available because of participant confidentiality and institutional/ethical restrictions, but data are available from the corresponding author upon reasonable request, subject to approval by the relevant institution and ethics committee. Requests to access the datasets should be directed to QV, bsquocbao@pnt.edu.vn.
