# Supplementary material for: Higher fibrosis-4 index is associated with 2-year cardiovascular events in Vietnamese adults with type 2 diabetes mellitus: a prospective cohort study
Source: Front Gastroenterol (Lausanne). 2026 Oct 7;5:1973311. doi: 10.3389/fgstr.2026.1973311 (PMC13683220; doi:10.3389/fgstr.2026.1973311)
Supplement: Supplementary file 1 [file DataSheet1.pdf]

Supplementary Material

Supplementary Methods S1. Liver measurement and sparse-event analysis

FibroScan© procedures followed the study protocol: fasting for at least 3 hours, right-lobe intercostal measurements with the M probe, at least 10 valid measurements, success rate ≥60%, and IQR/median <30% for CAP and LSM. The protocol-defined thresholds were established before the cardiovascular follow-up analyses and were unchanged from the earlier baseline liver-vascular report from the same research program (main manuscript reference 16). CAP categories were S0 ≤233, S1 234-269, S2 270-300, and S3 ≥301 dB/m; hepatic steatosis was therefore defined as CAP ≥234 dB/m. This threshold and grading scheme were retained for continuity with prior Vietnamese FibroScan work in adults with type 2 diabetes (main manuscript reference 15), rather than as a claim that 234 dB/m is a universal histologic cutoff. Protocol LSM categories were F0-1 <7.0 kPa, significant liver fibrosis ≥F2 at 7.0 to <8.7 kPa, advanced fibrosis ≥F3 at 8.7-11.5 kPa, and cirrhosis ≥11.5 kPa. The ≥7.0-kPa threshold for significant liver fibrosis was supported by biopsy-validated NAFLD transient-elastography data and was also used in the prior Vietnamese type 2 diabetes study (main manuscript references 15 and 19). Wong et al. identified 7.0 kPa as the best overall cutoff for ≥F2 and 8.7 kPa as the best overall cutoff for ≥F3; 11.5 kPa was a high-specificity threshold for cirrhosis.

Because expanded MACE (11 events) and conventional MACE (5 events) were sparse, Firth-penalized Cox regression was used for inferential models. Multivariable models were deliberately parsimonious. The core expanded-MACE model contained age and FIB-4; the main model added MAFLD status. Continuous FIB-4 hazard ratios were reported per 1-IQR increase using the FIB-4 distribution in the overall time-to-event cohort (n=280): Q1=0.939147, Q3=1.635313, and IQR=0.6961661 units. The same 0.6961661-unit scaling factor was used for the full cohort and all subgroup analyses so that effect estimates represent the same absolute change in FIB-4. Estimates were rescaled as HR_IQR = exp(beta x 0.6961661), equivalently HR_per-unit^0.6961661; confidence limits were transformed identically and p values were unchanged. The FIB-4 scaling factor was also fixed at the IQR of the original 280-participant analytic cohort (0.696 units) and was not recalculated after individual-event deletion, ensuring direct comparability of hazard ratios across leave-one-event-out models. LSM was evaluated both as a continuous variable and as LSM-defined significant liver fibrosis (≥7.0 kPa) in univariable Firth Cox models for expanded and conventional MACE. Sensitivity analyses omitted age, used conventional Cox estimation, examined LSM-defined significant liver fibrosis in place of FIB-4, assessed proportional-hazards assumptions with scaled Schoenfeld residuals, and explored time-varying FIB-4 effects when indicated.

Supplementary Methods S2. Bayesian model averaging and software reproducibility

BMA was prespecified as an exploratory model-uncertainty analysis for expanded MACE and was not used to select a single definitive model. Both BMA datasets included all 280 participants and all 11 expanded MACE. Two parallel candidate spaces were analyzed. The FIB-4 space contained age, sex, diabetes duration ≥10 years, hypertension, MAFLD, continuous FIB-4, HbA1c <7%, and LDL-C ≥2.6 mmol/L. The LSM space replaced FIB-4 with LSM-defined significant liver fibrosis. Each space therefore contained eight optional predictors and 256 possible predictor subsets.

Archived calls were: bic.surv.formula(f = Surv(time_month, mace_open) ~ age + gioi + dtd_10y + tha + fl + fib4 + a1c7 + ldl_26, data = d_bma_fib4, strict = FALSE, factor.type = TRUE) and bic.surv.formula(f = Surv(time_month, mace_open) ~ age + gioi + dtd_10y + tha + fl + f2_4 + a1c7 + ldl_26, data = d_bma_lsm, strict = FALSE, factor.type = TRUE).

The fitted BMA objects confirmed prior.param=0.5 for all eight predictors. For arguments not shown in the archived call, BMA 3.18.21 defaults were used: OR=20, maxCol=30, OR.fix=2, nbest=150, and factor.prior.adjust=FALSE. With strict=FALSE, models within the package's Occam-window criterion were retained. The final averaging sets contained 99 models in the FIB-4 space and 81 in the LSM space. Analyses were performed in R 4.6.0 with BMA 3.18.21, coxphf 1.13.4, and survival 3.8-9. PIPs

are posterior probabilities of inclusion within the specified model space and should not be interpreted as frequentist p values or as evidence of causality.

Supplementary Methods S3. Exploratory age-adjusted FIB-4 categories and piecewise time-varying analysis

For exploratory categorical survival analyses, FIB-4 was grouped using age-adjusted liver-fibrosis risk thresholds. For participants aged <65 years, low, intermediate, and high categories were <1.3, 1.3-2.67, and >2.67. For participants aged ≥65 years, the lower cutoff was increased to 2.0, yielding categories <2.0, 2.0-2.67, and >2.67. This age adjustment followed the principle in contemporary AASLD and EASL guidance that the lower FIB-4 threshold should be increased in older adults because age is embedded in the score (main manuscript references 3 and 4). These thresholds were developed for liver-fibrosis risk stratification, not cardiovascular prediction. The categorical analyses were therefore used here only as exploratory visualizations of the continuous FIB-4 association. In the full cohort, ordered groups were compared using a log-rank test for trend, and pairwise log-rank p values were reported descriptively. The same age-adjusted categories were also examined within the MAFLD subgroup using standard log-rank tests. The subgroup Kaplan-Meier analyses were considered exploratory because the high-risk MAFLD stratum contained only six participants and cardiovascular event counts were sparse.

For an additional exploratory assessment of possible non-proportionality, follow-up was split at 12 months. A counting-process dataset was created with one interval before and, when applicable, one interval after 12 months. The time-varying term was coded as FIB-4 after 12 months and 0 before 12 months, so the coefficient for FIB-4 represented the early-period slope and the added time-varying coefficient represented the change in slope after 12 months. The post-12-month FIB-4 slope is therefore the sum of these two coefficients. Age-adjusted Firth models were fitted in the full cohort and in the MAFLD subgroup. Profile-likelihood confidence intervals did not converge for all individual piecewise terms, reflecting the very small number of events in each period. Accordingly, the formal p value for the change term was emphasized; a derived post-12-month HR with a Wald confidence interval from the coefficient covariance matrix is supplied only as descriptive supplementary information. These analyses do not establish a 12-month biological change point.

Supplementary Table S1. Prespecified cardiovascular endpoint definitions and counting rule.

Endpoint	Operational definition
Cardiovascular death	Death from a definite or probable cardiovascular cause, including myocardial infarction, sudden cardiac death, heart failure, stroke, or another vascular cause.
Nonfatal myocardial infarction	Acute ischemic myocardial injury supported by compatible clinical features, electrocardiographic changes and a rise/fall in troponin with at least one value above the 99th percentile, according to the study definition.
Nonfatal stroke	Acute focal neurological dysfunction lasting >24 h with compatible ischemic or hemorrhagic brain imaging and no death.
Heart-failure hospitalization	Unplanned admission ≥24 h/overnight with heart failure as the principal diagnosis, objective symptoms/signs, compatible cardiac structural/functional abnormality, and intensification of heart-failure therapy.
Transient ischemic attack	Transient focal neurological symptoms attributed to cerebral, spinal, or retinal ischemia, lasting <24 h, without acute infarction on imaging, diagnosed by a neurologist.
Unstable-angina hospitalization	Hospitalization for rest or low-exertion ischemic chest pain without biomarker evidence of acute myocardial injury.

Endpoint	Operational definition
Peripheral arterial event or coronary/peripheral revascularization	Limb ischemia requiring invasive revascularization or amputation, or symptomatic chronic coronary disease treated with PCI/CABG, according to the study definition.
Time-to-first-event rule	A participant contributed one event at the first qualifying clinical episode. Multiple qualifying components within the same episode were not counted as separate events.

Potential events were verified from source documentation and confirmed by a physician in the relevant specialty. All but one qualifying event occurred at the study hospital. The single external episode (unstable angina treated with PCI and coronary stent) was verified with a discharge summary and written stent documentation; it was counted as one clinical event.

Supplementary Table S2. Bayesian model averaging settings and posterior inclusion probabilities.

Panel A. Model-space and software settings.

Setting	FIB-4 model space	LSM model space
Outcome	Expanded MACE, time to first event	Expanded MACE, time to first event
Participants / events	280 / 11	280 / 11
Liver fibrosis marker	FIB-4 (continuous)	Significant liver fibrosis (LSM ≥7.0 kPa)
Other candidate predictors	Age; sex; diabetes duration ≥10 y; hypertension; MAFLD; HbA1c <7%; LDL-C ≥2.6 mmol/L	Same
Optional predictors	8	8
Combinatorial subset space	256	256
Prior inclusion probability	0.50 for each predictor	0.50 for each predictor
strict	FALSE	FALSE
factor.type	TRUE	TRUE
OR (default)	20	20
maxCol (default)	30	30
OR.fix (default)	2	2
nbest (default)	150	150
factor.prior.adjust (default)	FALSE	FALSE
Models retained for averaging	99	81
BMA package	3.18.21	3.18.21

Panel B. Posterior inclusion probabilities (PIP, %).

Predictor	FIB-4 model space PIP (%)	LSM model space PIP (%)
Age	96.3	100.0
FIB-4	71.5	-
MAFLD	65.3	71.3
HbA1c <7%	58.3	52.3
LDL-C ≥2.6 mmol/L	39.3	37.7
Male sex	23.9	28.1

Predictor	FIB-4 model space PIP (%)	LSM model space PIP (%)
Diabetes duration ≥10 years	23.1	21.1
Hypertension	22.5	26.7
Significant liver fibrosis (LSM ≥7.0 kPa)	-	19.9

PIP, posterior inclusion probability; LSM, liver stiffness measurement. BMA was used to assess model uncertainty, not to choose the final Firth Cox model.

Supplementary Table S3. Full baseline characteristics by MAFLD status (n=284).

Characteristic	Overall	No MAFLD	MAFLD	p value
Age, years	59.1 +/- 10.7	60.8 +/- 10.5	58.5 +/- 10.7	0.109
Age ≥60 years, n (%)	154 (54.2)	46 (59.0)	108 (52.4)	0.323
Male sex, n (%)	121 (42.6)	39 (50.0)	82 (39.8)	0.121
BMI, kg/m2	24.7 +/- 3.7	23.1 +/- 2.8	25.3 +/- 3.8	<0.001
Overweight/obesity, n (%)	195 (68.7)	40 (51.3)	155 (75.2)	<0.001
Obesity, n (%)	116 (40.8)	15 (19.2)	101 (49.0)	<0.001
Abdominal obesity, n (%)	234 (82.4)	52 (66.7)	182 (88.3)	<0.001
Below recommended physical activity, n (%)	183 (64.4)	45 (57.7)	138 (67.0)	0.144
Known diabetes duration, years	6.0 (1.0-11.0)	6.5 (1.0-13.0)	5.0 (1.0-10.3)	0.299
Known diabetes duration ≥10 years, n (%)	97 (34.2)	31 (39.7)	66 (32.0)	0.222
Hypertension, n (%)	205 (72.2)	55 (70.5)	150 (72.8)	0.699
Blood pressure <130/80 mmHg, n (%)	228 (80.3)	66 (84.6)	162 (78.6)	0.259
Dyslipidemia, n (%)	262 (92.3)	66 (84.6)	196 (95.1)	0.003
Smoking, n (%)	42 (14.8)	16 (20.5)	26 (12.6)	0.095
Significant alcohol use, n (%)	34 (12.0)	10 (12.8)	24 (11.7)	0.786
Chronic hepatitis B, n (%)	27 (9.5)	6 (7.7)	21 (10.2)	0.521
Chronic hepatitis C, n (%)	7 (2.5)	3 (3.8)	4 (1.9)	0.398
HbA1c, %	7.0 (6.4-8.3)	6.9 (6.3-8.4)	7.1 (6.5-8.3)	0.416
HbA1c <7%, n (%)	124 (43.7)	38 (48.7)	86 (41.7)	0.290
LDL-C ≥2.6 mmol/L, n (%)	151 (53.2)	34 (43.6)	117 (56.8)	0.047
AST, U/L	26.2 (20.5-33.0)	25.8 (19.9-32.3)	26.3 (20.7-33.1)	0.754
ALT, U/L	27.8 (19.3-43.5)	24.7 (16.4-37.9)	28.9 (20.9-44.9)	0.028
Creatinine, micromol/L	81.5 (68.2-93.7)	86.7 (71.5-96.0)	78.6 (66.8-92.7)	0.050
Platelets, 10^9/L	254.3 +/- 65.7	256.1 +/- 75.2	253.9 +/- 62.0	0.776
FIB-4	1.2 (0.9-1.6)	1.3 (1.0-1.7)	1.2 (0.9-1.6)	0.642

Characteristic	Overall	No MAFLD	MAFLD	p value
CAP, dB/m	265.1 +/- 58.0	195.1 +/- 37.1	291.7 +/- 39.3	<0.001
Liver stiffness, kPa	5.5 (4.4-6.9)	4.8 (4.1-6.0)	5.7 (4.5-7.3)	0.001
Significant liver fibrosis, n (%)	68 (23.9)	12 (15.4)	56 (27.2)	0.038
SGLT2 inhibitor use, n (%)	15 (5.3)	4 (5.1)	11 (5.3)	1.000
RAS blocker use, n (%)	161 (56.7)	42 (53.8)	119 (57.8)	0.552
Statin use, n (%)	177 (62.3)	50 (64.1)	127 (61.7)	0.703
Aspirin use, n (%)	19 (6.7)	4 (5.1)	15 (7.3)	0.517

Values are mean +/- SD, median (interquartile range), or n (%). P values reproduce the thesis descriptive comparisons and are supplied only as supplementary detail; the main manuscript treats baseline characteristics descriptively.

Supplementary Table S4. Univariable Firth Cox regression for expanded MACE in the full cohort (FIB-4 effects per IQR [0.696-unit] increase).

Variable	HR	95% CI	p value
Age, per year	1.11	1.04-1.19	<0.001
Male sex	1.13	0.35-3.57	0.829
Diabetes duration ≥10 years	3.15	1.00-11.13	0.049
Hypertension	0.95	0.29-3.85	0.936
HbA1c <7%	0.34	0.06-1.20	0.096
LDL-C ≥2.6 mmol/L	0.32	0.08-1.04	0.059
FIB-4, per IQR (0.696-unit) increase	1.72	1.28-2.12	0.002
MAFLD	2.74	0.65-25.42	0.192
Liver stiffness, per 1-kPa increase	1.03	0.92-1.07	0.371
Significant liver fibrosis (LSM ≥7.0 kPa)	1.86	0.53-5.83	0.315

Supplementary Table S5. Leave-one-event-out sensitivity analyses of the FIB-4 association with cardiovascular events.

Omitted event	FIB-4 aHR per IQR	95% CI	p value
**Panel A. Expanded MACE**			
Event 1	1.60	1.11–2.05	0.019
Event 2	1.55	1.06–1.98	0.030
Event 3	1.63	1.14–2.10	0.013
Event 4	1.56	1.07–1.99	0.026
Event 5	1.60	1.13–2.02	0.014
Event 6	1.62	1.14–2.06	0.013
Event 7	1.58	1.09–2.02	0.021
Event 8	1.63	1.16–2.07	0.009
Event 9	1.57	1.08–2.02	0.025
Event 10	1.56	1.06–1.99	0.028
Event 11	1.22	0.54–2.40	0.600
**Full cohort**	**1.56**	**1.09–1.99**	**0.022**
**Panel B. Conventional MACE**			
Event 1	1.92	1.30–2.64	0.005
Event 2	1.86	1.26–2.47	0.006
Event 3	1.98	1.39–2.70	0.002
Event 4	1.82	1.21–2.41	0.010
Event 5	1.23	0.33–3.33	0.720
**Full cohort**	**1.81**	**1.24–2.39**	**0.007**

Note. Each refitted model sequentially omitted one participant with the relevant cardiovascular endpoint. Expanded-MACE models included age, FIB-4, and MAFLD; conventional-MACE models included age and FIB-4. FIB-4 was reported per the fixed overall-cohort IQR of 0.696 units. aHR, adjusted hazard ratio; CI, confidence interval; IQR, interquartile range; MACE, major adverse cardiovascular events.

Supplementary Table S6. Expanded MACE sensitivity and proportional-hazards results in the full cohort (FIB-4 effects per IQR [0.696-unit] increase where applicable).

Analysis / term	Estimate (95% CI)	p value / inference
Main Firth model: age	1.11 (1.03-1.19)	0.003
Main Firth model: FIB-4, per IQR	1.56 (1.09-1.99)	0.022
Main Firth model: MAFLD	3.51 (0.79-33.09)	0.105
Age-omitted Firth: FIB-4, per IQR	1.68 (1.27-2.07)	0.002
Age-omitted Firth: MAFLD	2.72 (0.64-25.23)	0.198
Conventional Cox: age	1.12 (1.04-1.20)	95% CI excludes 1
Conventional Cox: FIB-4, per IQR	1.49 (1.09-2.06)	95% CI excludes 1
Schoenfeld: age	-	0.578
Schoenfeld: FIB-4	-	0.012
Schoenfeld: MAFLD	-	0.953
Schoenfeld: global	-	0.096
FIB-4 x log(time/18 months)	-	0.025

Exact p values for the two conventional Cox sensitivity terms were not available in the reported analysis output; inference is therefore shown from the 95% confidence intervals only.

Supplementary Table S7. Exploratory 12-month piecewise Firth models for expanded MACE.

Population	Expanded MACE by period	Age term	FIB-4 0-12-month term	Added FIB-4 change after 12 months	Derived FIB-4 effect after 12 months
Full cohort (280 participants)	3 / 8	HR 1.105 (1.030-1.182); p=0.004	HR 0.74 per IQR; profile p=0.802	HR ratio 2.43 per IQR; profile p=0.317	HR 1.79 per IQR (Wald 95% CI 1.33-2.40); p<0.001
MAFLD subgroup (203 participants)	3 / 7	HR 1.150 (1.050-1.262); p=0.001	HR 0.47 per IQR; profile p=0.525	HR ratio 3.90 per IQR; profile p=0.169	HR 1.83 per IQR (Wald 95% CI 1.36-2.46); p<0.001

Events are shown as 0-12 months / after 12 months. All FIB-4 effect estimates in this table are expressed per the common overall-cohort IQR (0.6961661-unit) increase. The added FIB-4 term tests whether the FIB-4 slope changes after 12 months; its p value is therefore the formal test of a discrete change in effect. The derived post-12-month HR equals exp(beta_FIB-4 + beta_change) and its Wald confidence interval was calculated from the fitted covariance matrix. Profile-likelihood confidence intervals did not converge for all individual piecewise terms; therefore these estimates are descriptive sensitivity analyses only. The n values printed by coxphf for split datasets refer to observation intervals, not numbers of participants.

Supplementary Table S8. MAFLD subgroup analyses for expanded MACE (10 events; 392.0 person-years; FIB-4 effects per IQR [0.696-unit] increase).

Term	HR / aHR (95% CI)	p value
Univariable age, per year	1.15 (1.06-1.25)	<0.001
Univariable male sex	1.53 (0.45-5.15)	0.485

Term	HR / aHR (95% CI)	p value
Univariable diabetes duration ≥10 years	3.00 (0.91-10.82)	0.071
Univariable hypertension	0.81 (0.24-3.33)	0.747
Univariable LDL-C ≥2.6 mmol/L	0.35 (0.08-1.17)	0.089
Univariable HbA1c <7%	0.41 (0.08-1.48)	0.183
Univariable FIB-4, per IQR	1.73 (1.33-2.12)	<0.001
Univariable severe steatosis	1.00 (0.30-3.61)	0.999
Univariable liver stiffness, per 1-kPa increase	1.03 (0.89-1.11)	0.540
Univariable significant liver fibrosis	1.79 (0.49-5.90)	0.355
Adjusted age	1.13 (1.04-1.22)	0.002
Adjusted FIB-4, per IQR	1.63 (1.17-2.06)	0.009
Alternative model: significant liver fibrosis	1.01 (0.27-3.47)	0.994

Expanded-MACE incidence in this subgroup was 25.51 per 1,000 person-years (95% CI 12.23-46.91).

Supplementary Table S9. Exploratory cardiovascular events across age-adjusted FIB-4 categories.

Panel A. Event distribution and ordered log-rank trend.

Outcome	Low FIB-4	Intermediate FIB-4	High FIB-4	Log-rank trend chi-square	p value
Expanded MACE	5/191 (2.6%)	4/79 (5.1%)	2/10 (20.0%)	5.111	0.024
Conventional MACE	2/191 (1.0%)	2/79 (2.5%)	1/10 (10.0%)	3.253	0.071

Panel B. Pairwise log-rank comparisons (unadjusted p values).

Comparison	Expanded MACE p	Conventional MACE p
Low vs intermediate	0.326	0.374
Low vs high	0.004	0.024
Intermediate vs high	0.073	0.220

Age-adjusted categories: age <65 years, low <1.3, intermediate 1.3-2.67, high >2.67; age ≥65 years, low <2.0, intermediate 2.0-2.67, high >2.67. The log-rank statistic shown is the ordered trend test (1 df; trend weights -1, 0, 1). Pairwise p values are unadjusted and are descriptive only. The high category contained 10 participants, so all categorical comparisons are exploratory.

Supplementary Table S10. Univariable and adjusted Firth Cox analyses for conventional MACE in the full cohort (5 events; FIB-4 effects per IQR [0.696-unit] increase).

Term	HR / aHR (95% CI)	p value
Univariable age	1.10 (1.00-1.21)	0.046
Univariable male sex	0.96 (0.16-4.93)	0.964
Univariable diabetes duration ≥10 years	2.63 (0.51-15.76)	0.240
Univariable hypertension	1.17 (0.22-11.64)	0.868
Univariable HbA1c <7%	0.92 (0.15-4.72)	0.918

Term	HR / aHR (95% CI)	p value
Univariable LDL-C ≥2.6 mmol/L	0.26 (0.03-1.40)	0.121
Univariable FIB-4, per IQR	1.88 (1.33-2.43)	0.002
Univariable MAFLD	1.18 (0.22-11.77)	0.858
Univariable liver stiffness, per 1-kPa increase	1.05 (0.96-1.08)	0.155
Univariable significant liver fibrosis	4.28 (0.83-25.71)	0.080
Adjusted age	1.08 (0.98-1.19)	0.129
Adjusted FIB-4, per IQR	1.81 (1.24-2.39)	0.007

Supplementary Table S11. Conventional MACE analyses in the MAFLD subgroup (4 events; FIB-4 effects per IQR [0.696-unit] increase).

Term	HR / aHR (95% CI)	p value
Univariable male sex	1.54 (0.24-9.94)	0.633
Univariable age	1.13 (1.01-1.29)	0.030
Univariable hypertension	0.88 (0.14-9.03)	0.892
Univariable smoking	2.92 (0.28-17.73)	0.313
Univariable diabetes duration ≥10 years	2.06 (0.32-13.35)	0.423
Univariable LDL-C ≥2.6 mmol/L	0.32 (0.03-1.95)	0.219
Univariable HbA1c <7%	1.38 (0.21-8.93)	0.719
Univariable FIB-4, per IQR	1.94 (1.42-2.53)	<0.001
Univariable severe steatosis	1.61 (0.27-16.62)	0.615
Univariable liver stiffness, per 1-kPa increase	1.08 (0.95-1.16)	0.158
Univariable significant liver fibrosis	5.95 (0.98-61.40)	0.053
Adjusted age	1.11 (0.98-1.28)	0.101
Adjusted FIB-4, per IQR	1.87 (1.32-2.56)	0.003

All conventional-MACE analyses are exploratory because only four events occurred in this subgroup.

Supplementary Figures

Supplementary Figure S1. Scaled Schoenfeld residuals for FIB-4 in the full-cohort expanded-MACE conventional Cox model.

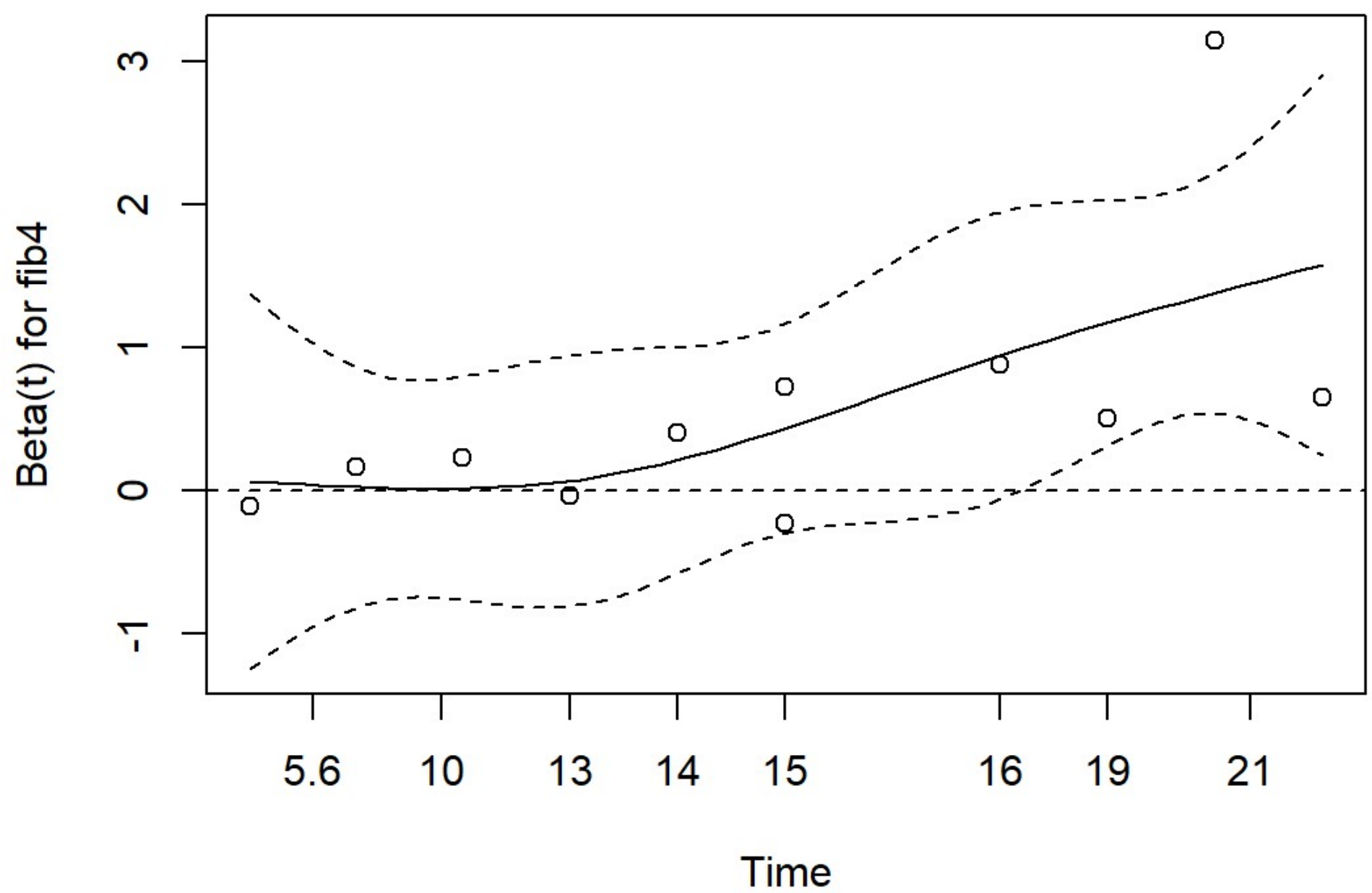

Supplementary Figure S2. Scaled Schoenfeld residuals for FIB-4 in the MAFLD-subgroup expanded-MACE conventional Cox model.

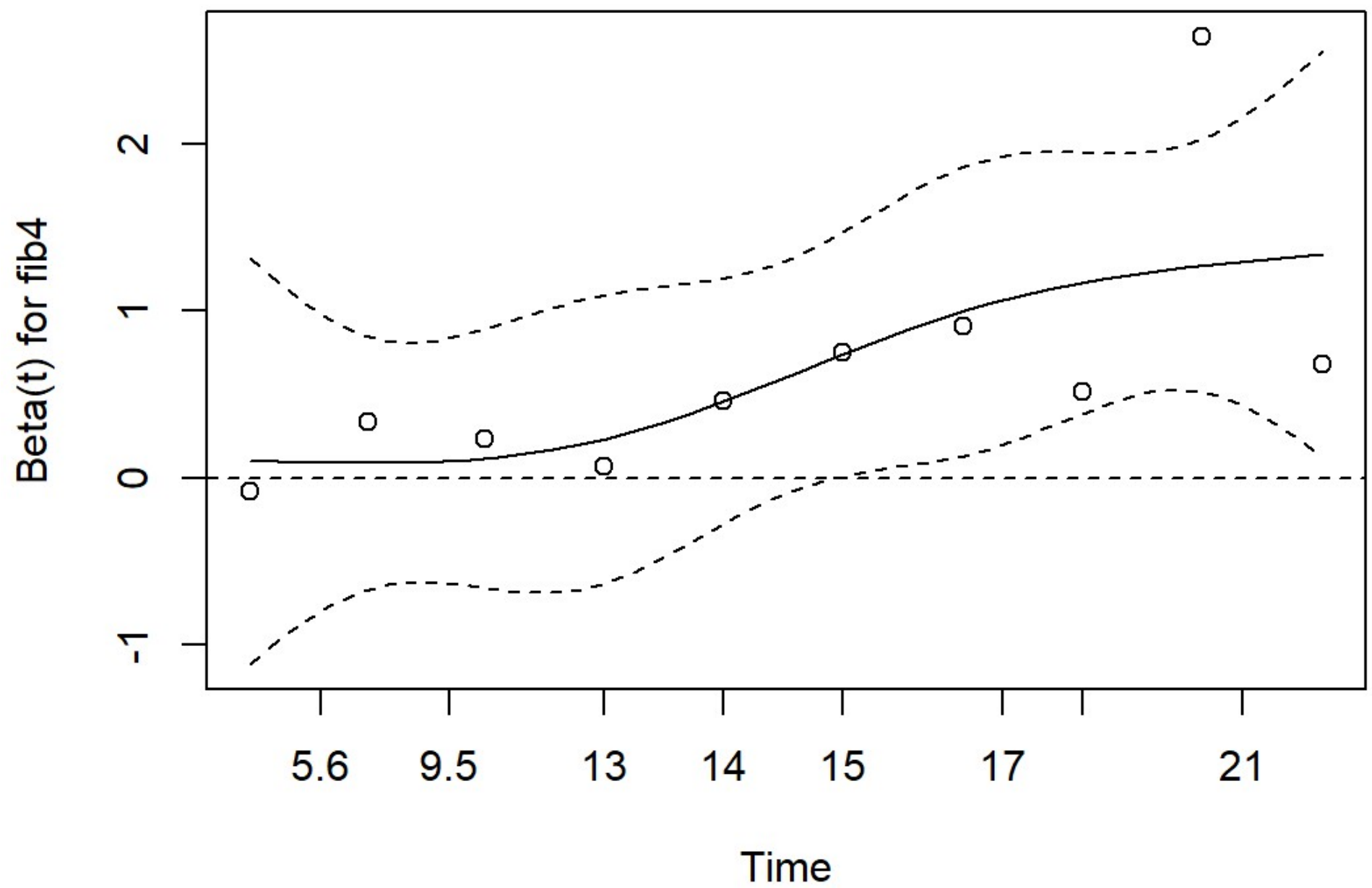

Supplementary Figure S3. Scaled Schoenfeld residuals for FIB-4 in the full-cohort conventional-MACE conventional Cox model.

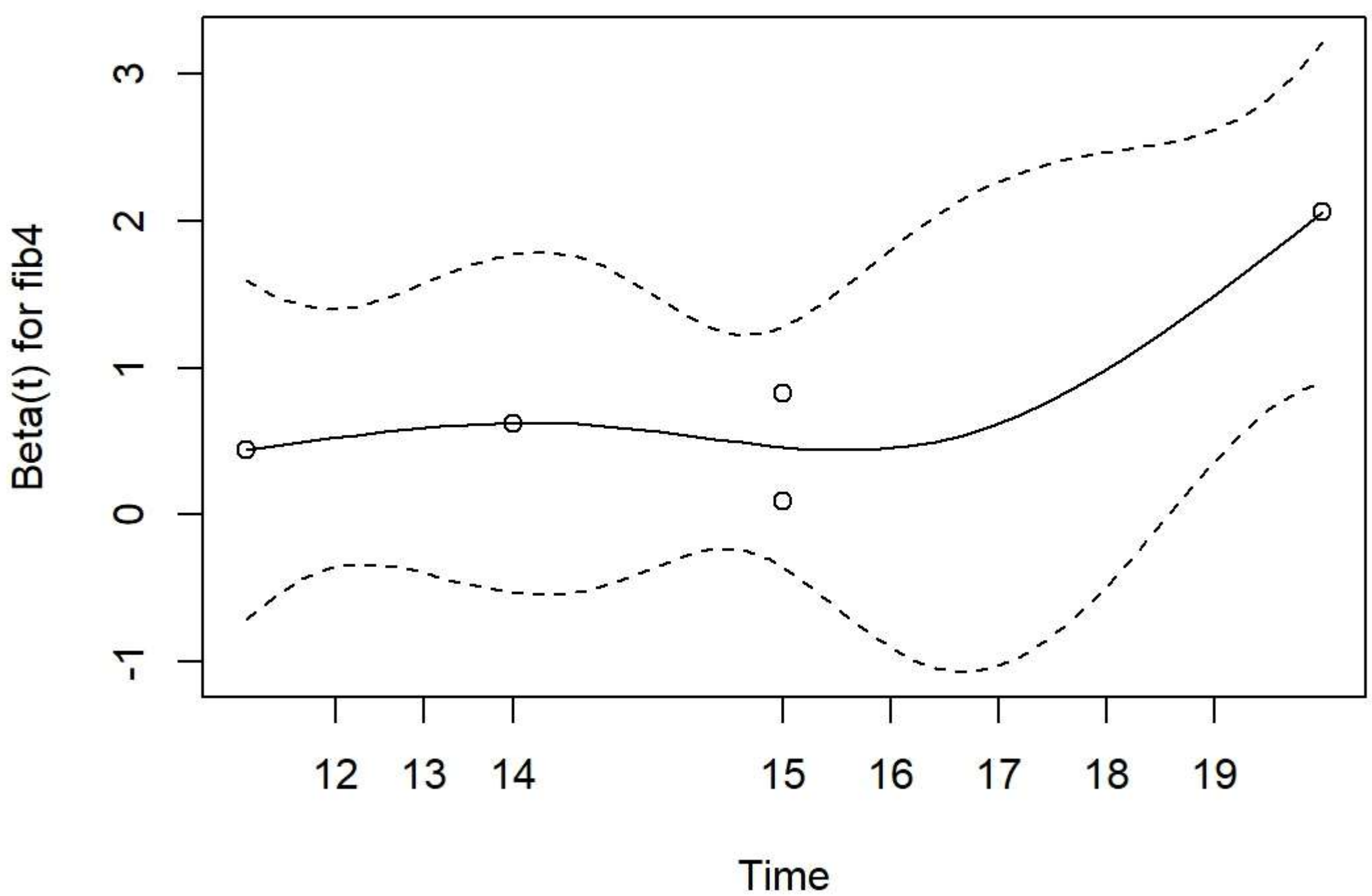

Supplementary Figure S4. Scaled Schoenfeld residuals for FIB-4 in the MAFLD-subgroup conventional-MACE conventional Cox model.

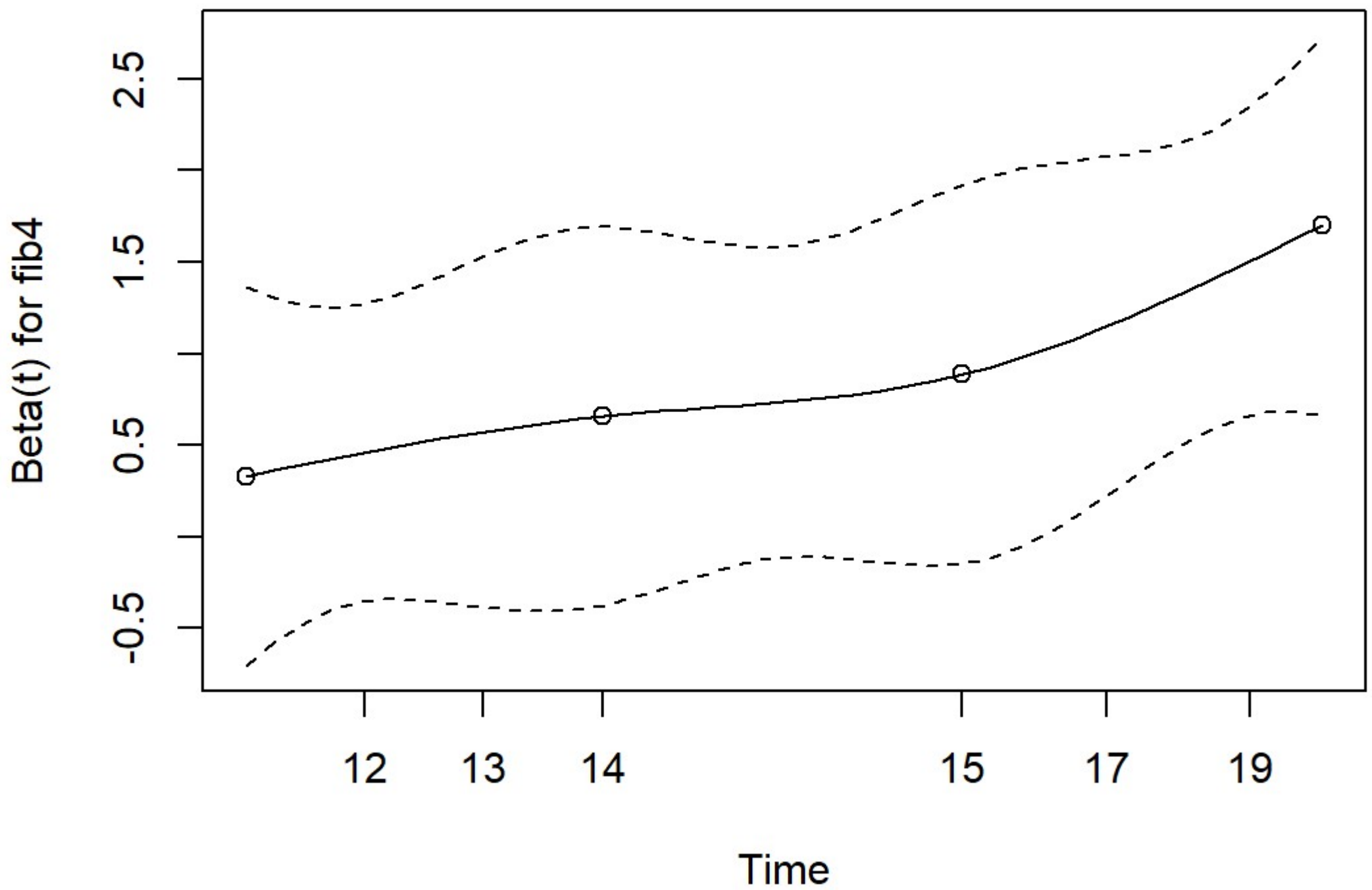

Supplementary Figure S5. Kaplan-Meier estimates of conventional-MACE-free survival across age-adjusted FIB-4 risk categories. Conventional MACE occurred in 2/191 (1.0%), 2/79 (2.5%), and 1/10 (10.0%) participants in the low-, intermediate-, and high-risk groups, respectively. The ordered log-rank test for trend did not reach statistical significance (chi-square=3.253, df=1; p=0.071). Age-adjusted category definitions were identical to those used for expanded MACE. Censoring marks and numbers at risk are shown; this analysis was exploratory because only five conventional MACE occurred.

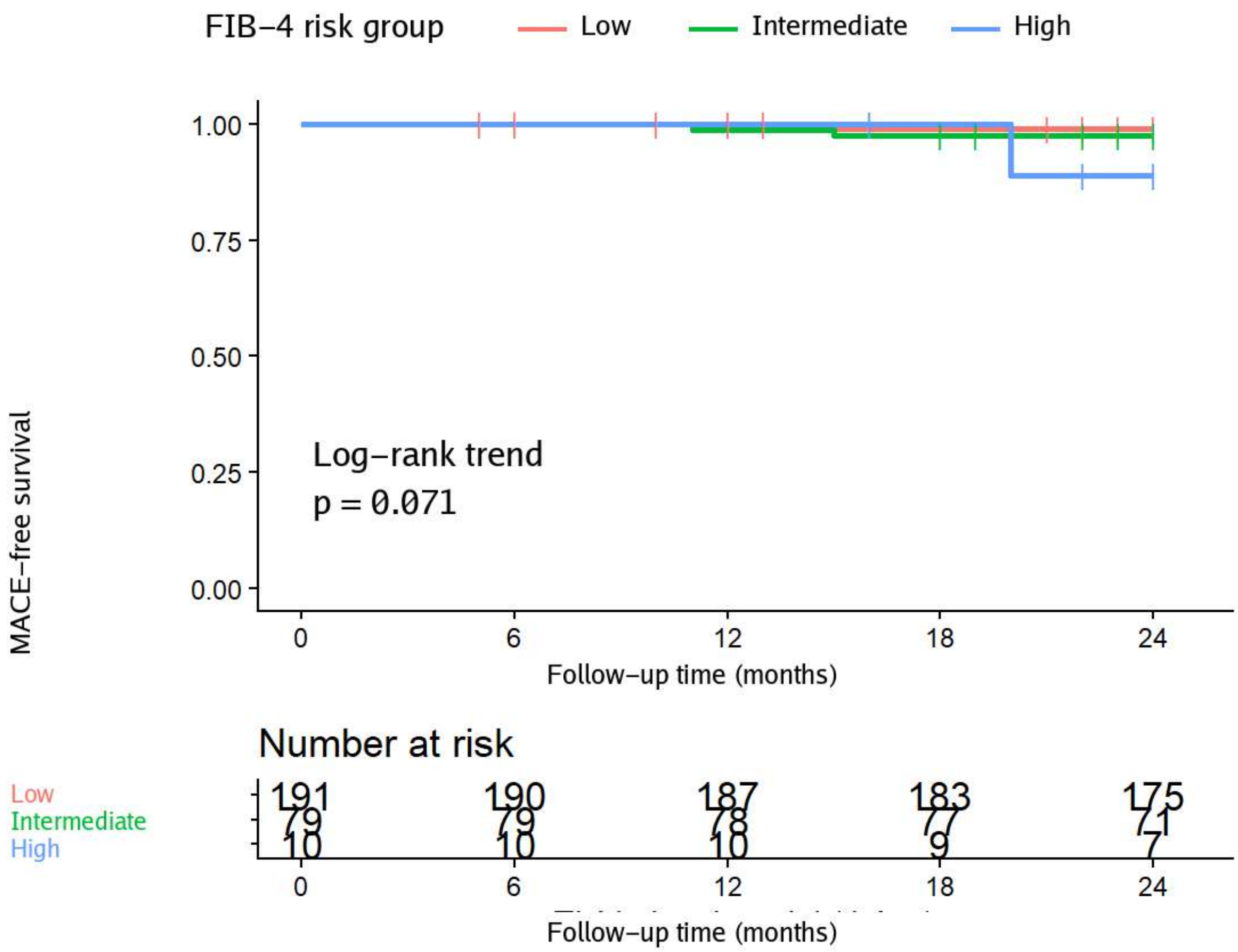

Supplementary Figure S6. Kaplan-Meier estimates of expanded-MACE-free survival across age-adjusted FIB-4 risk categories within the MAFLD subgroup (n=203). The low-, intermediate-, and high-risk strata contained 139, 58, and 6 participants, respectively. Survival distributions differed across the three categories (standard log-rank p=0.0026). Age-adjusted category definitions were identical to those used in the full cohort. Censoring marks and numbers at risk are shown. Because only six participants were in the high-risk stratum, this subgroup analysis was exploratory.

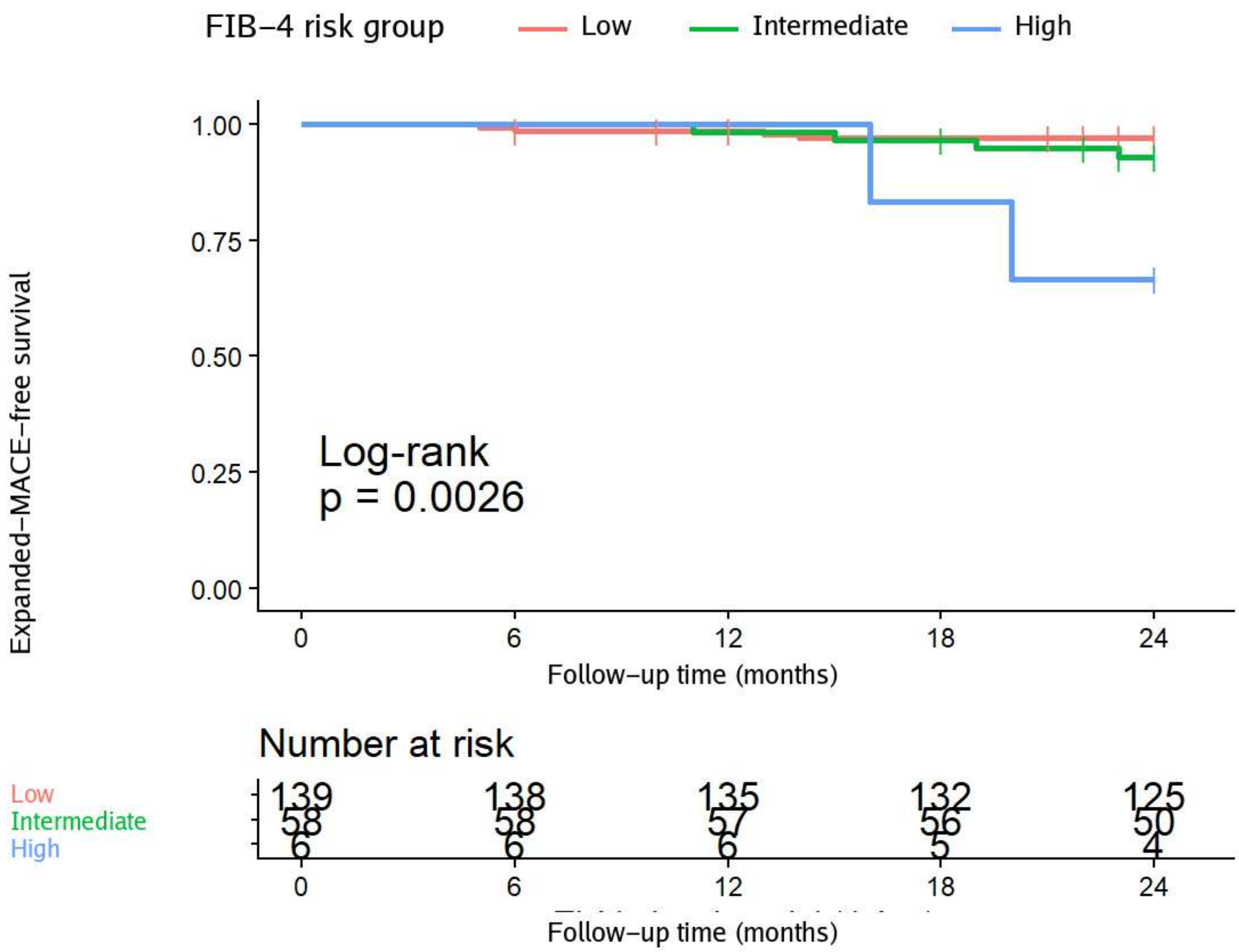

Supplementary Figure S7. Kaplan-Meier estimates of conventional-MACE-free survival across age-adjusted FIB-4 risk categories within the MAFLD subgroup (n=203). Survival distributions differed across the three categories (standard log-rank p=0.016). The low-, intermediate-, and high-risk strata contained 139, 58, and 6 participants, respectively, but only four conventional MACE occurred in the MAFLD subgroup. Age-adjusted category definitions were identical to those used in the full cohort. Censoring marks and numbers at risk are shown. This analysis was exploratory because of the very small event count and high-risk stratum.

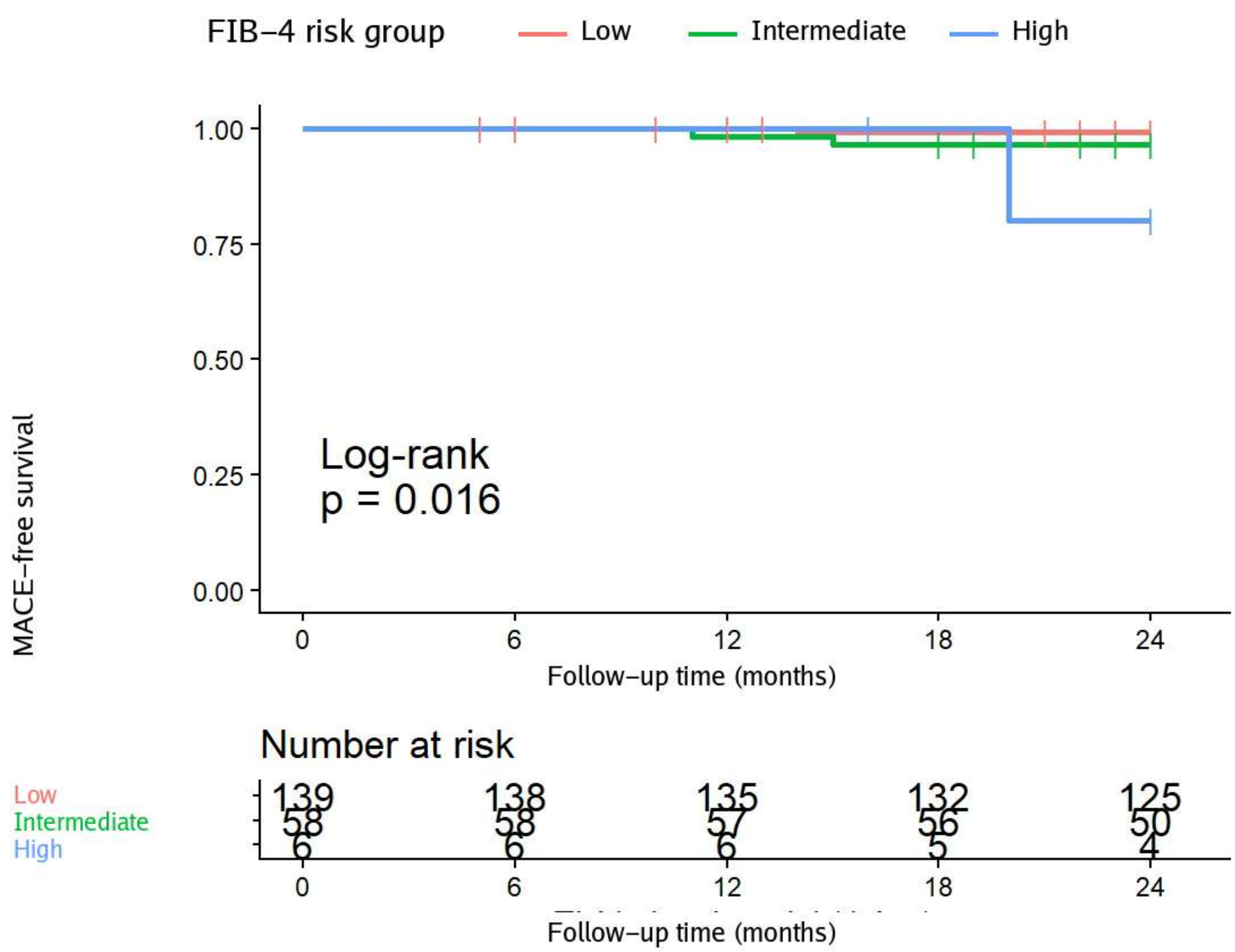

Supplementary Table S12. STROBE checklist

Item	Topic	Recommendation	Location
1	Title/abstract	Design and balanced summary	Title; Abstract
2	Background/rationale	Scientific background	Introduction
3	Objectives	Specific objectives	Introduction, final paragraph
4	Study design	Key design elements	Methods 2.1
5	Setting	Setting and dates	Methods 2.1
6	Participants	Eligibility, selection, follow-up	Methods 2.2; Figure 1
7	Variables	Outcome/exposure definitions	Methods 2.3-2.5; Table S1
8	Data sources/measurement	Sources and measurement methods	Methods 2.3-2.5; Supplementary Methods S1
9	Bias	Efforts to address bias	Methods; Discussion limitations
10	Study size	How the analytic cohort was assembled	Methods 2.1-2.2; Figure 1
11	Quantitative variables	Handling of quantitative variables	Methods 2.4, 2.6; Supplementary Methods S3
12	Statistical methods	Modeling, censoring, sensitivity analyses	Methods 2.6; Supplementary Methods S1-S3; Tables S2, S4-S7
13	Participants	Numbers at each stage	Results 3.1; Figure 1
14	Descriptive data	Characteristics and follow-up	Results 3.1; Table 1; Table S3
15	Outcome data	Events and rates	Results 3.2; Table 2
16	Main results	Adjusted estimates and precision	Results 3.3-3.5; Table 3; Tables S2, S4-S7
17	Other analyses	Subgroup/sensitivity analyses	Results 3.6-3.8; Tables S8-S11; Figures S1-S7
18	Key results	Summary relative to objectives	Discussion, paragraph 1
19	Limitations	Limitations and bias	Discussion, limitations paragraph
20	Interpretation	Cautious overall interpretation	Discussion
21	Generalisability	External validity	Discussion, final paragraph
22	Funding	Source and role of funders	Funding section
